# What Explains Cambodia’s Success in Reducing Child Stunting-2000-2014?

**DOI:** 10.1371/journal.pone.0162668

**Published:** 2016-09-20

**Authors:** Giacomo Zanello, C. S. Srinivasan, Bhavani Shankar

**Affiliations:** 1 School of Agriculture, Policy and Development, University of Reading, Reading, United Kingdom; 2 School of Agriculture, Policy and Development, University of Reading, Reading, United Kingdom; 3 Centre for Development, Environment and Policy, School of Oriental and African Studies, University of London, London, United Kingdom; The Hospital for Sick Children, CANADA

## Abstract

In many developing countries, high levels of child undernutrition persist alongside rapid economic growth. There is considerable interest in the study of countries that have made rapid progress in child nutrition to uncover the driving forces behind these improvements. Cambodia is often cited as a success case having reduced the incidence of child stunting from 51% to 34% over the period 2000 to 2014. To what extent is this success driven by improvements in the underlying determinants of nutrition, such as wealth and education, (“covariate effects”) and to what extent by changes in the *strengths of association* between these determinants and nutrition outcomes (“coefficient effects”)? Using determinants derived from the widely-applied UNICEF framework for the analysis of child nutrition and data from four Demographic and Health Surveys datasets, we apply quantile regression based decomposition methods to quantify the covariate and coefficient effect contributions to this improvement in child nutrition. The method used in the study allows the covariate and coefficient effects to vary across the entire distribution of child nutrition outcomes. There are important differences in the drivers of improvements in child nutrition between severely stunted and moderately stunted children and between rural and urban areas. The translation of improvements in household endowments, characteristics and practices into improvements in child nutrition (the coefficient effects) may be influenced by macroeconomic shocks or other events such as natural calamities or civil disturbance and may vary substantially over different time periods. Our analysis also highlights the need to explicitly examine the contribution of targeted child health and nutrition interventions to improvements in child nutrition in developing countries.

## Introduction

There is heightened international interest in reducing the burden of child undernutrition [1]. In 2012, the World Health Assembly unanimously agreed a set of six nutrition targets to be achieved by 2025, which included reduction in stunting in children under 5 years, reduction in the rate of infants born with low birth weight, reduction in the incidence of childhood wasting, increase in the rate of exclusive breastfeeding in the first six months and reduction in the rate of anaemia in women of reproductive age [2]. Stunting, derived from height-for-age measurements, captures the impacts of long term dietary inadequacy and infections, and is a key undernutrition metric in the international community [3]. The World Health Assembly Resolution set a 40% reduction in the number of children under-5 who are stunted as one of the six global nutrition targets for 2025 [2], a metric supported by the proposed Sustainable Development Goals [4]. Strong evidence suggests that stunting can have long-term effects on cognitive development, school achievement, economic productivity in adulthood and maternal reproductive outcomes [5–7] and it is a condition that may be very difficult to reverse [8]. Given the importance of growth in children in their first 1000 days, considerable research attention is being devoted to understanding the global distribution of height-for-age [1], the global drivers of inadequate height-for-age [3, 9], the burden of stunting and other measures of malnutrition [10] and interventions to effectively address them [11]. There is a strong rationale for investments in nutritional interventions, as averting stunting can produce life-long economic benefits [12].

In keeping with the notion of ‘positive deviance’ in nutrition, particular effort has been made to study countries that have made rapid progress in reducing stunting, and to uncover the driving forces behind these improvements. Headey [9] suggests a benchmark of one percentage point per year reduction in stunting or other anthropometric indicators over a sustained period to qualify as a ‘success’, and classifies and discusses the set of countries that meet the criterion. Individual country cases have also been examined. For example, O’Donnell et al. [13] examine Vietnam’s remarkable success in reducing stunting in the 1990s, and the extent to which this is explained by improvements in observed covariates of height-for-age, particularly income. Headey et al. [14] study the gains that Bangladesh has made in stunting reduction over fifteen years, and the roles played by each of a set of factors, such as parental education and improved sanitation.

Cambodia is a prominent success case, with stunting prevalence falling from an alarming 51.5% in 2000 to 34% in 2014. Some of this improvement is likely to have been spurred by the impressive economic progress achieved recently by Cambodia, with the poverty rate declining from 53% in 2004 to 22% in 2010 [15]. Gains in female education may have also contributed–the gender gap in schooling in Cambodia narrowed and closed over the last decade [15]. Improvements in drivers more proximal to child height, such as breastfeeding and antenatal care, may also have had a significant bearing on gains made in child height. Exclusive breastfeeding up to six months has been shown to be protective against several childhood infectious diseases [16]. In addition to reducing morbidity in children it may also serve as an indicator of practices such as complementary or overall infant feeding. Cambodia has recorded large improvements in breastfeeding indicators [17], underpinned by a government strategy that combined mass-media campaigns, training of health workers and community-based health initiatives [18]. The proportion of deliveries in healthcare facilities has gone up from 10% to 54% in the last decade, helped by public investments such as in a voucher scheme set up in 2007 to enable access to antenatal care [19]. Notwithstanding the observed remarkable improvement in child nutrition status, the annual burden of malnutrition in Cambodia was estimated to be US $ 400 million in 2010– nearly 2.5% of the Gross Domestic Product–based on an assessment of four discrete pathways of impact (1) mortality and disability in children with consequent lost value of a future workforce (2) child cognition deficit resulting in inferior school performance and adult productivity (3) current value of depressed productivity in working adults including tax losses for government and (4) current value of excess and preventable healthcare and welfare utilization [20]. It is, therefore, critical that improvements in child nutrition are sustained by effective policies and interventions.

## Previous Literature

Which of the several potential determinants of nutrition in Cambodia that have registered improvement in the period 2000–2014 have contributed most to the substantial reduction in stunting? A small literature has attempted to answer this question. Sunil and Sagna [21] use logistic regressions on DHS data from 2000 and 2005 to explain how proximal and socio-economic covariates are related to stunting status in Cambodia. They decompose the changes in the incidence of stunting to find that the improvements in proximate determinants, particularly prenatal care and breastfeeding and childhood vaccination were central to the decline in stunting prevalence. Similarly, Ikeda et al. [22] use logistic regressions to explain changes in stunting status using DHS data from 2000, 2005 and 2010 and find that the reduction in the incidence of stunting over this period was attributable to improvements in household wealth, sanitation, parental education, birth spacing and maternal tobacco use. Darapheak et al. [23] instead focused on children’s diet and found that stunting was negatively associated with dietary diversity, and in particular, that consumption of animal source food was a protective factor against stunting and underweight. Kov et al. [24] use regressions to explain variations in child HAZ using the 2005 and 2010 DHS datasets, with special focus on open defecation as an explanatory variable. They find that the significant change in open defecation that occurred in this period is able to explain much of the increase in mean child height.

This literature has increased understanding of the drivers of child height improvement in Cambodia. However, some important gaps remain in this understanding. Firstly, this literature has either dichotomized the continuous information on height-for-age Z scores (HAZ) into ‘stunted’ or ‘not-stunted’ status, or has modelled mean HAZ as a function of covariates. Whilst having the advantage of simplicity, these characterisations place strong restrictions on the way in which covariates are associated with the HAZ distribution. For example, dichotomisation would make no distinction between a mildly stunted child and a severely stunted one, while there could be a compelling case for a covariate such as breastfeeding to have different impacts on different parts of the HAZ distribution. The improvement in HAZ in Cambodia has been more pronounced in the lower tail of the distribution than in the middle. These factors argue for analysis that flexibly characterises the entire HAZ distribution and its relationship with covariates.

Secondly, the empirical models used in the literature do not account for changes in the quality of determinants over time. For instance, improvements in child HAZ scores may be influenced not only by the coverage of antenatal care but also by the quality of the care provided. We also show later in the paper that the impact of socio-demographic determinants on child nutrition status varies over time. In certain periods, improvements in socio-demographic determinants were associated with very modest improvement in child HAZ scores. This highlights the need to examine the factors which influence the translation of determinants into improved child nutrition outcomes. A related issue which has received relatively little attention in the literature is the contribution of targeted interventions which may not be reflected in the conventional determinants and the extent to which these conventional set of determinants included in empirical models can explain changes in child HAZ scores.

Thirdly, the literature has thus far pooled urban and rural areas of Cambodia in analysis, choosing to capture rural-urban differences by simple dummy variable characterisations (intercept shifters). However, the characterisation of rural and urban nutrition and their relationships with nutrition covariates can be quite different [25, 26]. This suggests the potential for important insights from separating analysis for urban and rural areas.

In this paper, we seek to contribute to the literature on explaining improvements in child height in Cambodia over time by applying recently developed methods that (i) break down the improvement into changes in covariates (“covariate effects”) and changes in the strength of relationship between covariates and height (“coefficient effects”) (ii) allow these covariate and coefficient effects to vary flexibly across the HAZ distribution. Thus, for example, we can examine the contribution of improvements in antenatal care coverage on height improvements, and also the contribution of improvements in the *quality* of antenatal care that may be reflected in coefficient changes. We treat urban and rural samples separately, allowing heterogeneous characterisation of their nutrition. We also assess the contribution of covariate effects and co-efficient effects separately over three time periods when the pace of change in HAZ scores appears to have been markedly different. Finally, we are able to broaden the previous timescale with the inclusion of a recently publicly made available DHS round of data from 2014. In the process, we highlight a methodology that holds substantial potential for further application in flexibly modelling the determinants of nutrition outcomes.

## Data and Methods

### Data

We have used datasets from the Demographic and Health Surveys of the MEASURE-DHS project (http://measuredhs.com/) which collects and disseminates nationally representative demographic, health and nutrition information based on household surveys for 90 countries. The datasets are freely accessible to the public and researchers subject to a prescribed registration and approval process. Permission to access and use the datasets relevant to this study was obtained by the authors from the MEASURE-DHS archive. For this study we used the data collected in Cambodia in 2000 (DHS IV round), 2004 (DHS V Round), 2010 (DHS VI round), and 2014 (DHS VII round). The samples are nationally representative and the units of observation used in this analysis are all children under the age of five years in the households surveyed. After a list wise deletion of observations with incomplete information, the sample for 2000 included 3446 children (86% rural and 14% urban), the sample for 2005 included 3459 children (80% rural and 20%), the sample for 2010 included 3623 children (74% rural and 26%) and the sample for 2014 included 4265 children (73% rural and 27% urban).

#### Outcome Variables

We used the child height-for-age Z scores (HAZ) as the indicator of child nutrition status in the analysis of the determinants of change in nutritional status over time and across rural and urban areas in Cambodia. Stunting, defined as HAZ less than two standard deviations of the NCHS/CDC/WHO International Reference Standard [27], is commonly used as an indicator of chronic nutritional deficiency that rarely can be reversed during the growth of children, with severe consequences for their health, learning process, and ultimately future earning opportunities. For all data sets, HAZ scores were computed using the 2006 WHO growth standards [27, 28].

#### Covariates

The conceptual framework underpinning our empirical analysis is the widely-applied UNICEF framework [29] outlining the causes of undernutrition. In the UNICEF framework, child malnutrition can be analysed in terms of immediate, underlying and basic causes. The immediate causes are inadequate dietary intakes and infectious disease, the underlying causes are inadequate maternal and child care, inadequate health services and health environment and the basic causes are institutional and socio-economic determinants and potential resources. The basic causes can be viewed as “exogenous” determinants–which influence child nutrition through their effect on the intervening proximate determinants. The proximate determinants are, therefore, endogenously determined by the exogenous characteristics. In empirical (reduced form) models examining the relationship between child nutrition outcomes and exogenous characteristics, the proximate determinants will generally be excluded to prevent biased and uninterpretable parameters [25,30]. Our empirical model, therefore, includes only the basic causes (socio-demographic determinants). However, in addition to the variables reflecting the basic causes, we also include covariates which are reliant mostly on exogenous public health provision rather than on socio-demographic endowments of the household. These include antenatal care, hospital births, breast feeding practices and vaccinations. Our argument for including these variables in the model is that changes in these variables are likely to be more responsive to policies, programmes and interventions rather than to changes in socio-demographic endowments of the household. For instance, it has been argued that policy, institutional and contextual settings are key determinants of the prevalence of breastfeeding practices [31].

The determinants of child health nutrition status used in this study are to a large extent based on the previous literature and include child and parental characteristics, maternal practices, socio-economic characteristics of the household and sanitation and water supply. Gender and age of the child are the child characteristics included in the model. Parental characteristics include maternal and paternal education (whether father and mother have received any school education), mother’s health, employment status of the mother (whether mother is currently working) and the dependency ratio calculated by dividing the number of inactive individuals (household members aged under 15 or over 64 years) by the active members in the household (aged 15 to 64 years). Maternal education has been consistently shown in the literature to have a positive association with child nutritional status [32]. The nutritional and health status of the mother–proxied by the Body Mass Index (BMI) of the mother—is controlled for as malnourished mothers may be more likely to deliver smaller babies and may be unable to provide adequate care to children [33, 34]. Mother’s employment may influence child nutrition status via the time that the mother is able to devote for child care [35], while the dependency ratio serves as an indicator of the overall burden of care incident on the parents [36]. Maternal practices included in the model cover whether the mother received prenatal visits, whether the birth took place in a hospital, whether the child was breastfed within one hour of birth and the vaccination status of the child (whether the child has received the recommended vaccinations for his/her age based on WHO guidelines [37]). Prenatal visits are important for dissemination of health information and also for preventive interventions [38], while deliveries at home may increase risks for both mother and child [39]. Breastfeeding is considered a key practice to support health, nutrition and development of children [10]. The socio-economic characteristics included in the model were a household wealth index (reflecting the asset endowments of the household) and access to sanitation and water supply. The wealth index is a composite measure based on the household’s ownership of assets and materials used for housing construction and is designed to capture the household’s cumulative living standard. Households with poorer endowments of assets may lack access to food and resources for care. The index is constructed using the same methods as used by the DHS [40] but it does not include the access to drinking water and sanitation facilities which are considered separately and it is constructed from the pooled dataset to make this measure comparable across the four survey rounds. We also included variables capturing whether the households have access to improved water and sanitation facilities [41] and the diffusion of improved sanitation facilities within the Primary Sampling Unit (PSU). The lack of access to safe water and sanitary waste disposal increases the diffusion of infectious diseases [42], and recent evidence has found a connection between child height and sanitation facilities at community level [43]. To capture regional effects related to the public health infrastructure or provision, we have grouped the provinces covered in the DHS datasets into for regions–South-East, South-West, North-East and North-West–with the South-East region (which includes the capital area of Phnom Penh) as the base-region.

### Methods

We first used ANOVA to test the differences between child HAZ scores and their determinants (child, parental and household characteristics) in 2000, 2004, 2010, and 2014 at the country level and separately for rural and urban populations. We then used a quantile regression (QR) based decomposition method to decompose the differences between child HAZ scores in different quantiles for each round of data into “covariate” and “co-efficient” contributions. When we are interested in examining the shifts in the distribution of the outcome variable (such as child HAZ scores) over time, a QR based approach [44] is preferable to mean or logistic regression based approaches because it allows the impact of the explanatory variables to vary along the entire distribution of the outcome variable. For instance, the impact of maternal education on child nutrition status may be very different in the lower quantiles of the child HAZ score distribution than in the upper quantiles, which would not be captured by using mean regression approaches. In analysing the contribution of determinants to child nutrition status, we are particularly interested in the lower end of the child HAZ score distribution characterised by severe stunting. Categorical dependent variables e.g., probit or logit models not only constrain the effect of explanatory covariates to be the same across the distribution of outcomes, these models sacrifice statistical information in grouping continuously distributed variables like HAZ into small numbers of categories. QR methods offer the most robust approach to flexibly model the shifts in HAZ distribution associated with changes to covariates.

The quantile regression method developed by Koenker and Bassett [44] estimates only the conditional quantile effects of changes in explanatory variables. In assessing the contribution of different determinants to changes in child nutrition status over time, we are more interested in the effect of a change in an explanatory variable (e.g., years of maternal education) in a population of individuals with different characteristics (unconditional effects) rather than in the impact for sub-groups with specific values of covariates (conditional effects). To assess the unconditional quantile effects of changes in explanatory variables, we use an unconditional Recentred Influence Function (RIF) QR regression method developed by Firpo et al. [45]. The RIF regression methods allow us to estimate the unconditional quantile effects of the covariates on child HAZ scores at any quantile of the HAZ distribution. All RIF quantile regressions were run using the household level sample weights provided in the DHS datasets. These sample weights are intended to make the sample nationally representative of the population.

Following Firpo et al. [45], the decomposition of the changes in child HAZ scores between each five year period 2000–2014, 2000–2005, 2005–2010, and 2010–2014 (separately for rural and urban areas) involves the following steps (the details of the decomposition method used are presented in S1 Appendix). First, we construct a counterfactual distribution of child HAZ scores which shows what the distribution of child HAZ scores would have been in *t*_1_ if the covariates in *t*_1_ had the same associations with HAZ (coefficients) as in *t*_0_. The counterfactual distribution is estimated using a probit-model based reweighting procedure developed by DiNardo et al. [46]. The difference between the distribution of the *t*_0_ HAZ scores and the counterfactual distribution gives the covariate effect and the difference between the counterfactual distribution and the distribution of *t*_1_ HAZ scores gives the coefficient effect. The RIF regression at different quantiles (which is estimated separately for the HAZ distribution of *t*_0_, *t*_1_ and the counterfactual distribution) is used in conjunction with the Blinder and Oaxaca [47] decomposition method to derive the contribution of individual covariates to the aggregate covariate and coefficient effects. The method adopted, therefore, allows us to decompose changes in child HAZ scores between *t*_0_ and *t*_1_ into components attributable to changes in the levels of the covariates and changes in the effects of the covariates. The analysis was carried out for the changes in the child HAZ score distribution between *t*_0_ and *t*_1_ for the country as a whole and also separately for rural and urban areas. In this paper we report only the decomposition results for rural and urban areas carried out separately.

#### Counterfactual Decompositions

The methodology adopted allows us to further decompose the changes in the child HAZ scores between 2000 and 2014 in terms of the contribution of individual covariates. For the detailed decomposition exercise we have grouped the large number of covariates into groups as shown in Table 1 in order to identify the key drivers of change in child nutrition status:

**Table 1:** Grouped covariates for counterfactual decompositions.

Grouped variable	Variables included
Child characteristics	• Gender of Child (0 = male, 1 = female) • Age of child
Maternal best practices	• Birth in hospital • Breastfeeding of child within one hour of birth • Child receiving recommended vaccinations (WHO norms) • Prenatal visits received by the mother
Parental characteristics	• Dependency ratio • Whether mother is currently working • Mother’s education (mother attended any school) • Father’s education (father attended any school) • Mother’s BMI (indicator of mother’s health)
Household wealth	• Assets index (constructed based on the ownership of a group of assets included in both surveys and normalised)
Sanitation and water supply	• Percent adoption of improved sanitation in the Primary Survey Unit (community) • Use of improved sanitation (UNICEF norms) • Use of improved drinking water (UNICEF norms)
Regional characteristics	• North-West region dummy • North-East region dummy • South-West region dummy

The contribution of the grouped variables can be interpreted as the combined effect of the constituent variables, except in the case of the regional characteristics group, which sums the effects of the individual regional dummies and reflects the overall impact of moving away from the base regions (south-east regions) and are somewhat difficult to interpret in the aggregate. For the detailed decomposition exercise, we focus on the lowest three quantiles (10^th^, 25^th^ and 50^th^), i.e., on the children most severely affected by chronic malnutrition.

## Results

### Descriptive Statistics

Table 2 provides descriptive statistics of changes in child nutrition outcomes in Cambodia between 2000 and 2014 for the whole country as well as separately for rural and urban areas. We also present the changes in HAZ scores across selected quantiles of the HAZ distribution. Table 3 summarises the changes in child HAZ scores between 2000 and 2014 at the mean and selected quantiles of the child HAZ score distribution for the country as a whole and separately for rural and urban areas. Table 4 shows the rural-urban differences in child HAZ scores at the mean and selected quantiles in the years 2000, 2005, 2010 and 2014.

**Table 2:** Changes in child HAZ scores in Cambodia between 2000 and 2014, at national and in rural and urban areas.

	National				Rural Areas				Urban Areas			
	2000	2005	2010	2014	2000	2005	2010	2014	2000	2005	2010	2014
Mean HAZ score	-1.897	-1.793	-1.664	-1.433	-1.926	-1.835	-1.739	-1.487	-1.726	-1.524	-1.260	-1.101
Percentage stunting	51.5%	46.3%	41.6%	33.9%	52.3%	47.5%	45.2%	36.5%	46.5%	41.6%	31.5%	27.0%
HAZ score by quantile												
10^th^ quantile	-3.91	-3.41	-3.26	-2.95	-3.90	-3.44	-3.35	-2.98	-3.95	-3.20	-2.88	-2.67
25^th^ quantile	-2.99	-2.61	-2.45	-2.24	-3.00	-2.64	-2.50	-2.28	-2.91	-2.41	-2.08	-1.96
50^th^ quantile	-1.99	-1.80	-1.73	-1.53	-2.04	-1.85	-1.81	-1.59	-1.70	-1.53	-1.35	-1.20
75^th^ quantile	-0.96	-1.01	-0.91	-0.70	-1.02	-1.06	-0.99	-0.77	-0.66	-0.68	-0.55	-0.28
90^th^ quantile	0.24	-0.17	-0.06	0.25	0.22	-0.24	-0.13	0.13	0.36	0.28	0.34	0.44

Source: Cambodia Demographic and Health Surveys of 2000, 2005, 2010, 2014. Statistics population weighted.

**Table 3:** Changes in national, rural and urban child HAZ scores in Cambodia between 2000 and 2014 by quantiles.

	National	Urban	Rural
Mean	0.46^‡^	0.44^‡^	0.63^‡^
10^th^ quantile	0.96^‡^	0.92^‡^	1.28^‡^
25^th^ quantile	0.75^‡^	0.72^‡^	0.95^‡^
50^th^ quantile	0.46^‡^	0.45^‡^	0.50^‡^
75^th^ quantile	0.26^‡^	0.25^‡^	0.38^‡^
90^th^ quantile	0.01	-0.09	0.08
Kolmogorov-Smirnov test	0.19^‡^	0.22^‡^	0.18^‡^

Note: The superscript symbol ‡ indicates changes significant at the 0.01 level respectively based on the Kolmogorov-Smirnov test for equality of child HAZ score distributions for 2000 and 2014. Source: Cambodia Demographic and Health Surveys of 2000 and 2014.

**Table 4:** Rural-Urban differences in child HAZ scores in Cambodia (2000–2014).

	2000	2005	2010	2014
Mean	-0.20^‡^	-0.32^‡^	-0.48^‡^	-0.39^‡^
10^th^ quantile	-0.05	0.24	0.47^‡^	0.31^‡^
25^th^ quantile	0.09	0.23^†^	0.42^‡^	0.32^‡^
50^th^ quantile	0.34^‡^	0.32^†^	0.46^‡^	0.39^‡^
75^th^ quantile	0.36^†^	0.38^†^	0.44^‡^	0.49^‡^
90^th^ quantile	0.14	0.52^‡^	0.47^‡^	0.31^‡^
Kolmogorov-Smirnov test	0.08^‡^	0.06^†^	0.16^‡^	0.14^‡^

Note: Differences in child HAZ scores computed as Rural child HAZ score–Urban child HAZ score at the mean and selected quantiles. The superscript symbols † and ‡ indicate differences significant at the 0.05 and 0.01 level respectively based the Kolmogorov-Smirnov test for equality of rural and urban child HAZ score distributions (unweighted samples). Source: Cambodia Demographic and Health Surveys of 2000, 2005, 2010, 2014.

Over the period under analysis, the national mean HAZ score has improved from -1.897 to -1.433 and the prevalence of child stunting has fallen quite sharply from 51.5% to 33.9%. The faster improvement seems to have occurred in the periods 2000–2004 and 2010–2014. The change in the national mean HAZ score, however, conceals a more complex picture of change in child nutrition outcomes across the distribution of child HAZ scores and between rural and urban areas. Across the child HAZ distribution, the largest improvements are seen in the lower half of the distribution (10^th^, 25^th^ and 50^th^ quantiles), while improvements in the top (75^th^ and 90^th^ quantiles) are modest (or negative) in some quantiles (see Table 3). Similarly, improvements in child HAZ scores over the period 2000–2014 are considerably higher for urban areas (0.625, 95% CI 0.492–0.758) compared to rural areas (0.439, 95% CI 0.377–0.501), translating into a larger decline in stunting in urban areas by 3.7 percentage points. The changes in the lower quantiles in urban areas are much larger than in rural areas (Table 4). These patterns of changes in child HAZ scores highlight the need to flexibly model the changes across the entire HAZ distribution rather than focusing on changes in the mean. These patterns may account for large changes in the percentage of children stunted being associated with relatively small changes in mean HAZ scores.

**Fig 1:**
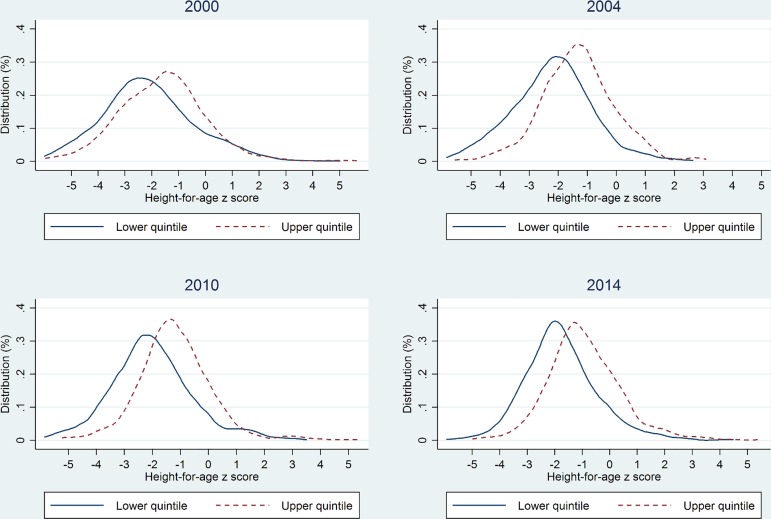
Changes in the gap between the poorest and the richest quintiles in the HAZ distribution of children aged less than 5 years according to four surveys, Cambodia 2000–2014.

Fig 1 compares the distribution of child HAZ scores of the lowest and highest income quintiles of households. The distributions of the lowest and highest income quintiles have come closer together over time indicating improvements in health equity. This also reflects the fact that the largest improvements in child HAZ scores have been seen in the lower half of the child HAZ score distribution.

Table 5 presents the characteristics of rural and urban areas of Cambodia from 2000 to 2014. A number of proximate determinants of child health have improved dramatically over this period. These include the proportion of children receiving basic vaccinations, proportion of children born in hospitals and proportion of mothers receiving pre-natal visits. Maternal education has also improved significantly, with the proportion of mothers with some school education increasing to 86.8% by 2014. Mother’s health status as indicated by BMI does show a greater change only in the last five years period. Access to improved sanitation and water supply has improved sharply in both rural and urban areas. The rural-urban differences in the changes in characteristics over 2000–2014 are summarised in Table 6. Percentage point improvements in urban areas are larger than in rural areas for vaccinations, maternal and paternal education, improved drinking water use and sanitation. The increase in the wealth index which is computed on the basis of the ownership of a group of assets has shown a larger increase in urban areas (1.67, 95% CI 1.55–1.79) than in rural areas (0.62, 95% CI 0.69–0.75). Improvements in rural areas are larger for hospital births and mothers receiving pre-natal visits although the levels of these determinants remain higher in urban areas. Improvement in the percentage of children breastfed within one hour of birth has been larger in rural areas and the level is also higher in rural areas.

**Table 5:** Characteristics of rural and urban areas in Cambodia, trends from 2000 to 2014.

	National				Rural				Urban			
	2000	2005	2010	2014	2000	2005	2010	2014	2000	2005	2010	2014
Children HAZ score	-1.90	-1.79	-1.66^‡^	-1.43^‡^	-1.93	-1.84	-1.74^‡^	-1.49^‡^	-1.73	-1.52	-1.26^‡^	-1.10^‡^
Gender (% of female children)	0.49	0.51	0.48	0.49	0.50	0.52	0.48	0.49	0.43	0.47	0.51	0.50
Age in months	30.04	29.66	29.79	28.99	29.92	29.59	29.91	29.04	30.74	30.09	29.12	28.67
Hospital birth (%)	0.11	0.22^‡^	0.53^‡^	0.85^‡^	0.07	0.18^‡^	0.47^‡^	0.83^‡^	0.36	0.47	0.85^‡^	0.97^‡^
Children breastfed within 1 hour of birth (%)	10.8%	36.4%^‡^	64.4%^‡^	65.2%^‡^	10.8%	36.3%^‡^	64.9%^‡^	66.7%	10.6%	37.1%^‡^	61.7%^‡^	55.9%^‡^
Mothers receiving pre-natal visits (%)	43.8%	60.1%^‡^	82.2%^‡^	90.5%^‡^	40.9%	58.7%^‡^	80.3%^‡^	89.7%^‡^	61.1%	69.0%^†^	92.8%^‡^	95.7%^‡^
Children receiving recommended vaccinations (WHO) (%)	33.4%	56.8%^‡^	67.2%^‡^	69.8%^‡^	30.9%	56.2%^‡^	66.2%^‡^	68.4%^‡^	48.0%	61.0%^‡^	72.4%^‡^	78.2%
Dependency ratio	1.42	1.25^‡^	1.10^‡^	1.01^‡^	1.43	1.27^‡^	1.13^‡^	1.03^‡^	1.35	1.14^‡^	0.93^‡^	0.84
Mother’s BMI	20.58	20.83^‡^	20.98^‡^	22.06^‡^	20.47	20.71^†^	20.83	21.96^‡^	21.20	21.61^†^	21.77	22.66^‡^
Mothers currently working (%)	70.6%	62.4%^‡^	64.4%^‡^	61.6%	72.2%	63.9%^‡^	66.0%^‡^	61.0%^†^	61.2%	52.9%^‡^	56.1%^‡^	65.1%^†^
Mothers attending any school (%)	67.6%	76.3%^‡^	81.0%^‡^	86.8%^‡^	66.3%	75.9%^‡^	78.8%^†^	85.4%^‡^	75.3%	79.2%	93.0%^‡^	95.2%
Fathers attending any school (%)	82.1%	85.5%	88.4%^‡^	89.3%	81.0%	85.0%	87.2%^‡^	88.1%	88.2%	88.8%	95.4%^‡^	96.3%
Continuous wealth index	-0.16	-0.11^‡^	0.11^‡^	0.56^‡^	-0.82	-0.34^‡^	-0.12^‡^	-0.10^‡^	-0.23	0.53^‡^	1.39^‡^	1.44^‡^
Improved water use (UNICEF definition) (%)	37.4%	40.3%^†^	51.8%^‡^	48.2%	35.8%	37.8%^†^	47.7%^‡^	45.5%	47.0%	55.8%	73.8%^‡^	64.9%^‡^
Improved sanitation use (UNICEF definition) (%)	15.6%	20.0%^‡^	34.6%^‡^	49.1%^‡^	10.4%	15.3%^‡^	25.9%^‡^	42.6%^‡^	46.4%	50.8%	81.4%^‡^	89.0%^‡^
Improved sanitation within the PSU (% households)	17.8%	20.0%	37.1%^‡^	49.1%^‡^	12.2%	15.3%	28.5%^‡^	42.6%^‡^	51.2%	50.8%	83.5%^‡^	89.0%^‡^
Sample size	3446	3459	3623	4265	2947	2764	2673	3104	499	695	950	1161

Note: The superscript symbols † and ‡ indicate change over the previous round significant at the 0.05 and 0.01 level respectively. Source: Cambodia Demographic and Health Surveys of 2000, 2005, 2010, 2014. Statistics population weighted.

**Table 6:** Rural-urban differences in changes in child and household characteristics in Cambodia from 2000–2014.

Characteristics	Change in rural areas (2000–2014)	95% CI for change in rural areas	Change in urban areas (2000–2014)	95% CI for change in urban areas	Difference (Urban–Rural)
Children HAZ score	0.44	0.363–0.515	0.63	0.45–0.80	0.19
Hospital birth (% point change)	76.0%	74.0%–77.2%	61.00%	57.2%–65.9%	-15.00%
Children breastfed within 1 hour of birth (% point change)	55.9%	53.9%–57.9%	45.30%	41.4%–49.3%	-10.60%
Mothers receiving pre-natal visits (% point change)	48.8%	46.7%–50.8%	34.60%	30.2%–39.1%	-14.20%
Children receiving recommended vaccinations (WHO) (% point change)	37.5%	35.1%–39.8%	30.20%	25.2% 35.2%	-7.30%
Dependency ratio	-0.40	-0.43 –-36.0	-0.51	-0.59–-0.43	-0.11
Mother’s BMI	1.49	1.338–1.637	1.46	1.147–1.763	-0.03
Mothers currently working (% point change)	-11.2%	-13.5%—-8.8%	3.90%	-1.2% -8.9%	15.10%
Mothers attending any school (% point change)	19.1%	16.9%–21.1%	19.90%	15.9%–23.9%	0.80%
Fathers attending any school (% point change)	7.1%	5.3% -8.9%	8.10%	5.0%–11.0%	1.00%
Continuous wealth index	0.72	0.69–0.75	1.67	1.55–1.79	0.95
Improved water use (UNICEF definition) (% point change)	9.7%	7.3%-12.2%	17.90%	12.8%–23.1%	8.20%
Improved sanitation use (UNICEF definition) (% point change)	32.2%	30.1%–34.2%	42.60%	37.9%–47.4%	10.40%
Improved sanitation within the PSU (% point change in households)	30.4%	29.0%–31.6%	37.80%	34.3%–41.4%	7.40%

### Unconditional RIF Regression Results

The estimates of the unconditional RIF quantile regressions—for each time period under analysis (2000–2014, 2000–2005, 2005–2010, and 2010–2014) are shown separately for urban and rural areas of Cambodia respectively for selected quantiles in S2 Appendix. The OLS estimates are also presented alongside for comparison. The RIF regression results highlight the potential importance as well as variability of coefficient effects, showing how the effects of determinants of child nutrition outcomes can vary over rural and urban areas, over time and across the child HAZ score distribution.

#### National level decompositions

The results of the national level decomposition of changes in child HAZ scores for over the period 2000–2014 are presented in Table 7 for the three lowest quantiles (the10^th^, 25^th^ and the 50^th^ quantiles). For each quantile, the observed change in child HAZ scores is decomposed into aggregate covariate and coefficient effects, which are further decomposed into explained and unexplained components. The explained components of the aggregate covariate and coefficient effects are decomposed into the contributions of the individual grouped covariates. At the national level, the covariate effects account for only 46–69% of the observed change in child HAZ scores. Household wealth and improved sanitation and water supply are the two significant drivers of improvements in child HAZ scores during this period. The child characteristics grouped variable is significant in all the quantiles; however, this variable only reflects the changing age and gender composition of children in the sample. The coefficient effects related to the covariates included in the model are not significant. However, the “unexplained” coefficient effects are very significant for the lowest (10^th^) quantile, which may be capturing the effects of unobserved variables, i.e., variables not included in the model.

**Table 7:** National decomposition of changes in child HAZ scores between 2000 and 2014.

	Quintile 1		Quintile 2		Quintile 3	
Observed HAZ Gap (A)	0.965***		0.744***		0.441***	
**Total Covariate Effect (B)**	0.440***	**(46%)**	0.356*	**(48%)**	0.305***	**(69%)**
*Explained (B1)*	*0*.383***	*(87%)*	*0*.382***	*(107%)*	*0*.308***	*(101%)*
Child characteristics	0.036**	(9%)	0.055***	(14%)	0.096***	(31%)
Maternal best practices	0.163	(43%)	0.102	(27%)	-0.002	(-1%)
Parental characteristics	0.056	(15%)	0.070*	(18%)	0.024	(8%)
Household wealth	0.051*	(13%)	0.084***	(22%)	0.105***	(34%)
Sanitation and water supply	0.069*	(18%)	0.084***	(22%)	0.097***	(31%)
Regional characteristics	0.007	(2%)	-0.013	(-3%)	-0.012	(-4%)
*Unexplained (B2)*	*0*.*057*	*(13%)*	*-0*.*026*	*(-7%)*	*-0*.*003*	*(-1%)*
**Total Co-efficient Effect (C)**	0.525***	**(54%)**	0.389*	**(52%)**	**0.136**	**(31%)**
*Explained (C1)*	*-0*.*094*	*(-18%)*	*0*.*130*	*(33%)*	*0*.*043*	*(32%)*
Child characteristics	-0.080	(85%)	-0.041	(-32%)	-0.041	(-95%)
Maternal best practices	-0.025	(27%)	0.014	(11%)	-0.002	(-5%)
Parental characteristics	0.057	(-61%)	0.078	(60%)	-0.003	(-7%)
Household wealth	-0.063	(67%)	0.065	(50%)	0.153*	(356%)
Sanitation and water supply	-0.004	(4%)	0.015	(12%)	-0.033	(-77%)
Regional characteristics	0.021	(-22%)	-0.003	(-2%)	-0.032	(-74%)
*Unexplained (C2)*	*0*.618***	*(118%)*	*0*.*259*	*(67%)*	*0*.*094*	*(69%)*

Note: The observed HAZ gap is decomposed into covariate effect and co-efficient effect (A = B + C). Each effect is in turn separated into explained and unexplained components (respectively B = B1+B2 and C = C1+C2). The explained effects (B1 and C1) are broken down further into the contributions of the grouped variables. Percentages for each grouped variable capture the relative contribution of the variable to the total explained covariate/coefficient effect. Asterisks show level of significance *** = significant at 1% level, ** = significant at 5% level and * = significant at 10% level. Estimations population weighted.

#### Decompositions for rural and urban areas

The results of the detailed decomposition exercise for rural areas are presented in Table 8. The decomposition exercise has been carried out separately for three time periods– 2000–2005, 2005–2010 and 2010–2014 –for the three lowest quantiles (10^th^, 25^th^ and 50^th^). For all the three quantiles, there was a strong improvement in child HAZ scores in the period 2000–2005, followed by a sharp slowdown in the period 2005–2010 and recovery in the period 2010–2014. Covariate effects made a large contribution to improvements in child HAZ scores in all the three time periods, but in the period 2000–2005, the positive covariate effects appear to have been offset by large negative and significant coefficient effects. When we consider the covariate effects of the individual grouped covariates, we find that improvements in parental characteristics (which reflect improvements in maternal and paternal education) is significant in all the quantiles and time periods, making a positive contribution to improvement in child HAZ scores. Improvements in sanitation and water supply are significant and make a large contribution to improvements in child HAZ scores in 2005–2010 and 2010–2014 in the 25^th^ and 50^th^ quantiles. Similarly, improvements in maternal best practices are significant in 2000–2005 in the 25^th^ and 50^th^ quantiles. Household wealth is not a significant contributor to improvements in child HAZ scores in the bottom quantile but is significant and positive in the 25^th^ and 50^th^ quantiles. Regional characteristics are significant in many of the quantiles and time periods. While the aggregate coefficient effects were significant and negative in the period -2005-2010, they were significant and positive in 2000–2005 and 2010–2014. Among the grouped covariates, the coefficient effects of maternal best practices were significant in the period 2000–2005 in the 10^th^ and 25^th^ quantiles. Coefficient effects related to wealth and parental characteristics have been positive and strongly significant in the 25^th^ and 50^th^ quantiles in the period 2010–2014.

**Table 8:** Rural areas: 5-year decomposition of changes in child HAZ scores (2000–2014).

	Quintile 1						Quintile 2						Quintile 3					
	2000–2005		2005–2010		2010–2014		2000–2005		2005–2010		2010–2014		2000–2005		2005–2010		2010–2014	
Observed HAZ Gap (A)	0.490***		0.093		0.357***		0.364***		0.134**		0.201***		0.198***		0.022		0.233***	
**Total Covariate Effect (B)**	0.279***	**(57%)**	0.330***	**(355%)**	0.167***	**(47%)**	0.247***	**(68%)**	0.298***	**(222%)**	0.165***	**(82%)**	0.170***	**(86%)**	0.198***	**(900%)**	0.125***	**(54%)**
*Explained (B1)*	*0*.212***	*(76%)*	*0*.404***	*(122%)*	*0*.113***	*(68%)*	*0*.191***	*(77%)*	*0*.263***	*(88%)*	*0*.151***	*(92%)*	*0*.123***	*(72%)*	*0*.184***	*(93%)*	*0*.109***	*(87%)*
Child characteristics	0.016**	(8%)	-0.003	(-1%)	0.005*	(4%)	0.019**	(10%)	-0.006	(-2%)	0.006**	(4%)	0.014*	(11%)	-0.007	(-4%)	0.011**	(10%)
Maternal best practices	0.022	(10%)	0.189***	(47%)	0.032	(28%)	0.033	(17%)	0.126***	(48%)	0.062*	(41%)	-0.015	(-12%)	0.074**	(40%)	0.026	(24%)
Parental characteristics	0.067**	(32%)	0.063***	(16%)	0.032**	(28%)	0.055**	(29%)	0.027**	(10%)	0.036***	(24%)	0.026	(21%)	0.022**	(12%)	0.023**	(21%)
Household wealth	0.025	(12%)	0.025	(6%)	0.019	(17%)	0.050***	(26%)	0.035***	(13%)	0.028***	(19%)	0.032**	(26%)	0.029**	(16%)	0.023**	(21%)
Sanitation &water supply	0.014	(7%)	0.078**	(19%)	0.026	(23%)	0.011	(6%)	0.053***	(20%)	0.035**	(23%)	0.016**	(13%)	0.046***	(25%)	0.036**	(33%)
Regional characteristics	0.068***	(32%)	0.051**	(13%)	-0.001	(-1%)	0.023	(12%)	0.028**	(11%)	-0.016	(-11%)	0.049***	(40%)	0.019	(10%)	-0.010	(-9%)
*Unexplained (B2)*	*0*.*066*	*(24%)*	*-0*.*074*	*(-22%)*	*0*.*055*	*(33%)*	*0*.*056*	*(23%)*	*0*.*035*	*(12%)*	*0*.*014*	*(8%)*	*0*.*047*	*(28%)*	*0*.*014*	*(7%)*	*0*.*017*	*(14%)*
**Total Co-efficient Effect (C)**	0.211**	**(43%)**	-0.237***	**(-255%)**	0.190**	**(53%)**	**0.117**	**(32%)**	-0.164**	**(-122%)**	**0.036**	**(18%)**	**0.029**	**(15%)**	-0.176***	**(-800%)**	0.108**	**(46%)**
*Explained (C1)*	*-0*.249***	*(-118%)*	*-0*.068*	*(29%)*	*0*.*020*	*(11%)*	*-0*.*021*	*(-18%)*	*-0*.064**	*(39%)*	*0*.063**	*(175%)*	*-0*.*032*	*(-110%)*	*-0*.*044*	*(25%)*	*0*.082***	*(76%)*
Child characteristics	-0.004	(2%)	0.001	(-1%)	0.000	(0%)	-0.007	(33%)	0.000	(0%)	-0.000	(0%)	-0.004	(13%)	0.000	(0%)	-0.000	(0%)
Maternal best practices	-0.003	(1%)	-0.023*	(34%)	-0.005	(-25%)	-0.003	(14%)	-0.017*	(27%)	0.004	(6%)	-0.002	(6%)	-0.006	(14%)	0.003	(4%)
Parental characteristics	-0.047**	(19%)	-0.021	(31%)	0.015	(75%)	-0.031*	(148%)	-0.028*	(44%)	0.037***	(59%)	-0.014	(44%)	-0.012	(27%)	0.034***	(41%)
Household wealth	-0.028	(11%)	-0.000	(0%)	0.015	(75%)	0.065	(-310%)	-0.001	(2%)	0.027***	(43%)	0.043	(-134%)	-0.001	(2%)	0.036***	(44%)
Sanitation and water supply	-0.000	(0%)	0.006	(-9%)	0.009	(45%)	-0.004	(19%)	-0.005	(8%)	0.006	(10%)	-0.002	(6%)	-0.002	(5%)	0.006	(7%)
Regional characteristics	-0.167***	(67%)	-0.032	(47%)	-0.015	(-75%)	-0.040	(190%)	-0.013	(20%)	-0.011	(-17%)	-0.053**	(166%)	-0.022	(50%)	0.003	(4%)
*Unexplained (C2)*	*0*.460***	*(218%)*	*-0*.168*	*(71%)*	*0*.170**	*(89%)*	*0*.*137*	*(117%)*	*-0*.*099*	*(60%)*	*-0*.*027*	*(-75%)*	*0*.*061*	*(210%)*	*-0*.132**	*(75%)*	*0*.*025*	*(23%)*

Note: The observed HAZ gap is decomposed into covariate effect and co-efficient effect (A = B + C). Each effect is in turn separated in explained and unexplained components (respectively B = B1+B2 and C = C1+C2). The explained effects (B1 and C1) are broken down further into contributions of the grouped variables. Percentages for each grouped variable capture the relative contribution of the variable to the total explained covariate/coefficient effect. Asterisks show level of significance *** = significant at 1% level, ** = significant at 5% level and * = significant at 10% level. Estimations population weighted.

The results of the decomposition exercise for urban areas are presented in Table 9. The two lower quantiles (10^th^ and 25^th^) witnessed a remarkable improvement in child HAZ scores in 2005, followed by a significant slowdown in 2005–2010, which has been further accentuated in the period 2010–2014. Covariate effects have been the significant and dominant drivers of improvements in child HAZ scores in all quantiles and time periods. In urban areas, household wealth is the only covariate that has consistently made a significant and positive contribution in all quantiles and time periods. Covariate effects of maternal best practices are significant in 2000–2005 in the 25^th^ and 50^th^ quantiles, but improvements in sanitation and water supply are generally not significant. The aggregate coefficient effects are generally not significant except in the 10^th^ quantile in 2000–2005, where there is a large significant coefficient effect associated with the “unexplained” component.

**Table 9:** Urban areas: 5-year decomposition of changes in child HAZ scores (2000–2014).

	Quintile 1						Quintile 2						Quintile 3					
	2000–2005		2005–2010		2010–2014		2000–2005		2005–2010		2010–2014		2000–2005		2005–2010		2010–2014	
Observed HAZ Gap (A)	0.726***		0.329**		0.212*		0.476***		0.341***		0.107		0.137		0.234**		0.121	
**Total Covariate Effect (B)**	0.386***	**(53%)**	**0.145**	**(44%)**	0.204**	**(96%)**	0.389***	**(82%)**	0.212**	**(62%)**	0.154**	**(144%)**	0.314***	**(229%)**	0.258***	**(110%)**	0.117*	**(97%)**
*Explained (B1)*	*0*.417***	*(108%)*	*0*.265**	*(183%)*	*0*.148**	*(73%)*	*0*.488***	*(125%)*	*0*.314***	*(148%)*	*0*.170**	*(110%)*	*0*.439***	*(140%)*	*0*.321***	*(124%)*	*0*.132**	*(113%)*
Child characteristics	0.013	(3%)	0.005	(2%)	0.000	(0%)	0.017	(3%)	0.003	(1%)	-0.006	(-4%)	0.017	(4%)	0.012	(4%)	-0.015	(-11%)
Maternal best practices	0.106	(25%)	0.103	(39%)	-0.060	(-41%)	0.127*	(26%)	0.051	(16%)	0.021	(12%)	0.164***	(37%)	0.062	(19%)	0.034	(26%)
Parental characteristics	0.128*	(31%)	0.052	(20%)	0.010	(7%)	0.104*	(21%)	0.038*	(12%)	0.017	(10%)	0.003	(1%)	0.018	(6%)	0.019	(14%)
Household wealth	0.142**	(34%)	0.124*	(47%)	0.072**	(49%)	0.213***	(44%)	0.142***	(45%)	0.130***	(76%)	0.202***	(46%)	0.124**	(39%)	0.116***	(88%)
Sanitation & water supply	0.039	(9%)	0.018	(7%)	0.138**	(93%)	0.075*	(15%)	0.049	(16%)	0.026	(15%)	0.020	(5%)	0.112	(35%)	-0.010	(-8%)
Regional characteristics	-0.012	(-3%)	-0.038	(-14%)	-0.012	(-8%)	-0.049	(-10%)	0.030	(10%)	-0.018	(-11%)	0.033	(8%)	-0.007	(-2%)	-0.011	(-8%)
*Unexplained (B2)*	*-0*.*031*	*(-8%)*	*-0*.*119*	*(-82%)*	*0*.*056*	*(27%)*	*-0*.*099*	*(-25%)*	*-0*.*102*	*(-48%)*	*-0*.*015*	*(-10%)*	*-0*.*125*	*(-40%)*	*-0*.*063*	*(-24%)*	*-0*.*015*	*(-13%)*
**Total Co-efficient Effect (C)**	0.340*	**(47%)**	**0.184**	**(56%)**	**0.008**	**(4%)**	**0.087**	**(18%)**	**0.129**	**(38%)**	**-0.048**	**(-45%)**	**-0.177**	**(-129%)**	**-0.025**	**(-11%)**	**0.004**	**(3%)**
*Explained (C1)*	*-0*.246**	*(-72%)*	*0*.*092*	*(50%)*	*-0*.*042*	*(-525%)*	*-0*.*153*	*(-176%)*	*0*.093*	*(72%)*	*-0*.*002*	*(4%)*	*-0*.*065*	*(37%)*	*0*.125***	*(-500%)*	*-0*.*025*	*(-625%)*
Child characteristics	-0.023	(9%)	0.012	(13%)	0.005	(-12%)	-0.020	(13%)	0.012	(13%)	0.010	(-500%)	-0.013	(20%)	0.008	(6%)	0.017	(-68%)
Maternal best practices	-0.007	(3%)	0.010	(11%)	-0.002	(5%)	0.035	(-23%)	0.019	(20%)	0.016	(-800%)	0.025	(-38%)	0.006	(5%)	-0.011	(44%)
Parental characteristics	0.025	(-10%)	0.056	(61%)	-0.026	(62%)	-0.037	(24%)	0.051	(55%)	-0.013	(650%)	0.038	(-58%)	0.050*	(40%)	-0.018	(72%)
Household wealth	-0.002	(1%)	0.017	(18%)	-0.038	(90%)	-0.022	(14%)	0.010	(11%)	-0.042	(2100%)	-0.030	(46%)	0.003	(2%)	-0.037	(148%)
Sanitation & water supply	-0.174**	(71%)	0.011	(12%)	-0.022	(52%)	-0.107*	(70%)	-0.002	(-2%)	0.028	(-1400%)	-0.031	(48%)	0.043	(34%)	0.044*	(-176%)
Regional characteristics	-0.065	(26%)	-0.015	(-16%)	0.041	(-98%)	-0.001	(1%)	0.002	(2%)	-0.001	(50%)	-0.053	(82%)	0.016	(13%)	-0.019	(76%)
*Unexplained (C2)*	*0*.586***	*(172%)*	*0*.*092*	*(50%)*	*0*.*050*	*(625%)*	*0*.*240*	*(276%)*	*0*.*036*	*(28%)*	*-0*.*045*	*(94%)*	*-0*.*113*	*(64%)*	*-0*.*150*	*(600%)*	*0*.*028*	*(700%)*

Note: The observed HAZ gap is decomposed into covariate effect and co-efficient effect (A = B + C). Each effect is in turn separated in explained and unexplained components (respectively B = B1+B2 and C = C1+C2). The explained effects (B1 and C1) are broken down further into contributions of the grouped variables. Percentages for each grouped variable capture the relative contribution of the variable to the total explained covariate/coefficient effect. Asterisks show level of significance *** = significant at 1% level, ** = significant at 5% level and * = significant at 10% level. Estimations population weighted.

## Discussion

Cambodia is regarded as one of the developing countries that have been successful in significantly reducing the incidence of child malnutrition since 2000 [48]. While Cambodia’s performance appears to be impressive in relation to the very high levels of stunting prevalent at the baseline, it clearly has a long way to go. 32% of children in Cambodia still remain stunted [49] in spite of the annual GDP growth rates of around 7% per annum since 2002 [50]. Our results are consistent with the findings from the previous literature [21, 22] that determinants derived from the UNICEF framework (parental education, maternal practices, sanitation and water supply and public health provision) make a major contribution to improvements in child nutrition status. However, the analysis in this paper shows that the reduction in the incidence of stunting in Cambodia over the decade is underlain by a more complex picture of change where the role of the key determinants of child nutrition outcomes varies substantially across the child HAZ distribution and over time and the drivers of change are quite different between rural and urban areas.

Our analysis suggests that determinants that impact the nutrition status of the most severely stunted children may be different from those that influence the nutrition status of moderately stunted children. Our results show that for children in the lowest quantiles of the HAZ distribution in rural areas–the most severely stunted children–maternal best practices and parental characteristics (incorporating parental education levels) are the most important drivers of changes in child nutrition status, with wealth making a much smaller contribution. For the most severely stunted children in urban areas, maternal best practices and parental characteristics are important drivers, but household wealth endowments play a larger role in improving child nutrition status. Improvement in health infrastructure–principally the use of improved sanitation and drinking water–plays an important role only in the upper quantiles, i.e., for moderately stunted children. The small contribution of gender and age composition of children to the changes in nutrition status suggests discrimination against female children may not be a major issue of concern in Cambodia.

An important insight from our analysis is that the conventional determinants included in empirical models of nutrition may not fully explain the observed changes in the incidence of child stunting. Our results highlight the need to examine factors that influence the translation of these determinants into improvements in child nutrition. Macroeconomic developments and disruption caused by natural calamities may have a large influence on how household endowments are translated into nutritional outcomes. The slowdown in improvements in child HAZ scores during the period 2000–2005 was the result of positive covariate effects (improvements in socio-demographic determinants) being offset by negative coefficient effects. Major factors that may explain the negative coefficient effects over this period include the global financial crisis from 2008–2009, the sharp rise in food prices over the same period and damage caused by recurrent floods in Cambodia. There is a considerable body of evidence which shows the severe adverse effects of the global financial crisis and the sharp increase in food prices from 2008–09 on child nutrition and education and general poverty in Cambodia and other developing countries [51–56]. The Cambodia Anthropometric Survey of 2008 [57] found an alarming increase in wasting in poor urban children from 6% in 2005 to 15.9% in 2008 (a level that was said to constitute a “humanitarian emergency”) owing to a sharp rise in food prices. Similarly, the adverse impacts of recurrent floods on child nutrition and health via loss of crops, damage to public health and education infrastructure and reduced access to sanitation and drinking water supply have been extensively documented [55,58]. The explanatory variables derived from the UNICEF framework may not capture the effects of macroeconomic or natural calamity shocks. For instance, asset-based household wealth indices may not reflect the impact of rising prices on access to food. The decomposition method adopted in this paper allows macroeconomic and other shocks to influence the translation of household endowments and characteristics into child nutrition impacts via the coefficient effects.

While the contribution of the conventional determinants to improvements in child nutrition may be modulated by co-efficient effects as discussed above, the large “unexplained” coefficients, especially in the bottom quantile in rural areas may be attributable to measurement errors in the variables or picking up the impacts of determinants not included in the model. The unexplained component may be principally attributable to the impact of targeted interventions. Over the past decade Cambodia has undertaken several targeted child health and nutrition programmes. The National Nutrition Programme in Cambodia has identified three priority areas for interventions (1) multiple micronutrient powders (2) complementary feeding and (3) management of acute malnutrition. The current Micronutrient Interventions Programme covers anaemia prevention and control, multiple micronutrient supplementation programme, the national vitamin A programme and the national iodine deficiency disorder programme. The Infant and Young Child Feeding Programmes include the Baby Friendly Hospital Initiative (BFHI), the Baby Friendly Community Initiative (BFCI) and management of acute malnutrition. While some of the interventions have been implemented on a national scale, others have been implemented on a much smaller scale. The interventions implemented on a large scale include vitamin A supplementation for children 6–59 months and postpartum mothers, iron and folic acid supplementation for pregnant and postpartum women, breastfeeding promotion and salt iodisation. The smaller scale interventions include complementary feeding promotion, multiple micronutrient powders for children of 6–24 months, weekly iron/folic acid supplementation for women of reproductive age, BFHI and BFCI which have been implemented in a limited number of provinces or in a selected number of hospitals and health centres [59]. The coverage achieved by the national scale interventions appears to be impressive. The coverage of vitamin A supplementation for children under 5 years increased from 29% to 71% between 2000 and 2010 while coverage of children aged 1–5 years for deworming increased from 30% to 60% over the same period. Similarly, the percentage of mothers receiving micronutrient supplementation and deworming increased from around 10–20% to 55–80% over the period 2000–2009. The percentage of children breastfed within a day of birth increased from 25% in 2000 to 89% in 2010, while children 0–5 months exclusively breastfed rose from 11%–74% over the same period. Household coverage of iodised salt also increased from 14% to 83% over this period [60, 61] although it has been noted that the quality of iodised salt products has deteriorated over the period 2008–2014 [62]. The dramatic improvements in the coverage of children and mothers under these initiatives suggest that interventions taken up on scale have been fairly well targeted at the most deprived children. The contributions of the targeted interventions to improvements in child HAZ scores may be masked if the improvements are attributed to the conventional determinants alone. Our analysis provides a starting point for the systematic consideration of the role of targeted interventions.

### Limitations

The analysis in this paper relies on data from four rounds of the Demographic and Health Survey for Cambodia. As with most large scale surveys in developing countries, the DHS also faces challenges in identifying a truly nationally representative sample (e.g., problems may arise if poor households do not appear in local authority household lists) and has to address biases arising from changes in the questionnaire used in different rounds of the survey. To obtain a nationally representative sample, the DHS independently prepares household lists in each Enumeration Area (EA) selected. There are also issues that arise from changes in the structure or wording of the survey questionnaires over the different rounds of DHS. Conkle (2007) has pointed out that the dramatic improvements in exclusive breastfeeding practices observed between 2000 and 2005 may be attributable to biases introduced due to changes to the wording of questions between 2000 and 2005 and seasonality factors [63]. Nevertheless, the DHS data sets provide robust and reliable measures of changes in the nutrition and health parameters and their determinants. A large proportion of observations had to be excluded from the analysis on account of missing data on child HAZ scores or other covariates. The deletion of observations with incomplete information could potentially bias our estimates if the missing observations are systematically related to certain household characteristics (e.g., if most of the observations with missing information are from poorer households). However, we found no significant differences in the characteristics of households with and without missing information. Details of missing observations for child HAZ scores and other variables in each of the DHS datasets used in the analysis and comparison of characteristics of all sample households and the sub-sample of households with complete information is presented in S3 Appendix.

While the method adopted in this paper allows the decomposition of changes in child HAZ scores into covariate and coefficient effects, it does not tell us what the factors driving the coefficient effects are–whether these are related to the quality of determinants, macroeconomic developments or other external shocks. These factors need to be explored separately, as nutrition and health data sets often have no data on these factors (e.g., food prices, improvements/deterioration in public health provision or access to services for different groups). Similarly, while the decomposition exercise accommodates the contribution of unobserved determinants (or determinants not included in the model), it does not identify these determinants. However, the quantification of the contribution of observed determinants and its decomposition into covariate and coefficient effects should prompt a systematic examination of the unobserved determinants and the factors that influence the translation of household endowments and characteristics into child nutrition outcomes.

## Conclusions

The framework for the analysis of child nutrition in terms of immediate, underlying and basic causes has underpinned most empirical studies on improvements in child nutrition in developing countries. However, to understand the child nutrition transitions in developing countries, the determinants derived from this framework need to be modelled flexibly, allowing their impacts to vary across the distribution of child nutrition outcomes, over time and between rural and urban areas. A flexible modelling approach can provide a more nuanced understanding of child nutrition transitions, as the important drivers for change may be different for severely stunted children and moderately stunted children and for rural and urban areas. The returns to the conventional determinants–the translation of household endowments, characteristics and practices into gains in child nutrition–may be influenced by macroeconomic factors and shocks. The conventional determinants may not fully or adequately explain changes in child nutrition in developing countries, which highlights the need to explicitly consider the role of targeted child health and nutrition interventions. The main insights from the analysis can inform the design of policy and programme interventions. Nutrition interventions need to focus on the determinants that have the largest impact in the lower quantiles of the child HAZ score distribution to reduce the incidence of stunting. Further, the nature of interventions may need to be different in rural and urban areas. Maternal best practices and parental education appear to be the most promising areas for intervention in rural areas, whereas improvements in socio-demographic endowments like wealth may produce a larger impact in urban areas. Importantly, policy makers need to consider the efficacy with which conventional determinants translate into improvements in child HAZ scores and on the macro-economic developments and public health infrastructure that influence this efficacy. While targeted child nutrition interventions may be making a larger contributions to reduction in stunting, there is very limited data at present on the coverage, quality and impacts of these programmes in most developing countries. Empirical impact assessments of these interventions should be a priority area for future research.

## Supporting Information

S1 Appendix Counterfactual Decomposition Procedure Using Unconditional Recentred Influence Function (RIF) quantile regression. (DOCX)

S2 Appendix Unconditional RIF regression results for rural and urban Cambodia. (DOCX)

S3 Appendix Demographic and Health Surveys for Cambodia–Observations with missing data. (DOCX)
