# Supplementary material for: What Explains Cambodia’s Success in Reducing Child Stunting-2000-2014?
Source: PLoS One. 2016 Sep 20;11(9):e0162668. doi: 10.1371/journal.pone.0162668 (PMC5029902; doi:10.1371/journal.pone.0162668)
Supplement: S1 Appendix — (DOCX) [file pone.0162668.s001.docx]

**S1 Appendix: Counterfactual Decomposition Procedure Using Unconditional Recentred Influence Function (RIF) quantile regression**

The counterfactual decomposition of differences in the distribution of child HAZ scores was carried out separately for the years 2000-2014, and 2000-2005, 2005-2010, 2010-2014 for both rural and urban areas of Cambodia. To assess the differences in the distribution of child HAZ scores between $t_{1}$ and $t_{2}$, we first estimate the distributions of HAZ scores for each area separately for $t_{1}$ and $t_{2}$ using kernel smoothing techniques. From the kernel density estimates of HAZ scores, the differential between HAZ scores in $t_{1}$ and $t_{2}$ is computed at each quantile and provides the raw difference in HAZ scores across the distribution.

Following Firpo et al. [1], the decomposition of differences between $t_{1}$ and $t_{2}$ HAZ scores (for each area, i.e., rural and urban) proceeds in two steps. In the first step, a counterfactual distribution of $t_{2}$ HAZ scores is constructed using a reweighting procedure suggested by Di Nardo et al. [2]; this is the distribution of HAZ scores in $t_{2}$ areas that would have prevailed if the households in the $t_{2}$ sample had the same returns to their characteristics as households in the $t_{1}$ sample. If *q (HAZ_t_1_)* and *q (HAZ_t_2_)* are given quantiles of the HAZ score distribution in $t_{1}$ and $t_{2}$, and *q (HAZ_C_)* is the same quantile of the counterfactual $t_{2}$ distribution, then the overall difference between $t_{2}$ and $t_{1}$ HAZ scores at any given quantile can be decomposed as:

*q (HAZ_t_2_)* - *q (HAZ_t_1_)* = [*q (HAZ_t_2_)* - *q (HAZ_C_)*] + [*q (HAZ_C_)* - *q (HAZ_t_1_)*] (1)

where [*q(HAZ_C_)* - *q(HAZ_t_1_)*] represents the covariate effect and [*q(HAZ_t_2_)* - *q(HAZ_C_)*] represents the coefficient effect. In the second step, the covariate and coefficient effects are each decomposed into the contribution of individual covariates using the Recentred Influence Function (RIF) regression [3] to obtain unconditional quantile effects of covariates on HAZ scores. The RIF regression is of the form:

*E (RIF (HAZ|q_τ_) = Xβ* (2)

where β represents the unconditional effect of covariate X on quantile τ of HAZ scores. The RIF unconditional regressions are separately estimated for $t_{1}$, $t_{2}$ and counterfactual HAZ score distributions:

$\hat{RIF}\left( {HAZ}_{K},\hat{q_{\tau}} \right)= X_{K}\hat{\beta_{K}}$ (3)

where K indexes the $t_{1}$, $t_{2}$ or the counterfactual sample. Using the RIF unconditional quantile estimates the following decomposition can be obtained for any given quantile:

$\hat{q_{\tau}}\left( {HAZ}_{t\_2} \right)- \hat{q_{\tau}}\left( {HAZ}_{t\_1} \right)=\left[ \bar{X_{t\_2}}\left( \hat{\beta_{C}} \right.-\left. \hat{\beta_{t\_2}} \right)+ \hat{R^{Coeff}} \right]+\left[ \left( \bar{X_{t\_1}}\hat{\beta_{t\_1}}-\bar{X_{t\_2.}}\hat{\beta_{C}} \right)+\hat{R^{Cov}} \right]$ (4)

where $\hat{q_{\tau}}\left( {HAZ}_{t\_2} \right)- \hat{q_{\tau}}\left( {HAZ}_{t\_1} \right)$represents the raw difference in $t_{2}$ and $t_{1}$ HAZ scores at the τth quantile and X represents the covariate averages. Note that $\hat{\beta_{C}}$is estimated from an RIF regression of the counterfactual HAZ score distribution. $\left( \hat{\beta_{C}} \right.-\left. \hat{\beta_{R}} \right)$ is, therefore, the difference in the effects of covariates between rural and urban areas and $\bar{X_{t\_2}}\left( \hat{\beta_{C}} \right.-\left. \hat{\beta_{R}} \right)$ represents the coefficient effect. $\left( \bar{X_{t\_1}}\hat{\beta_{t\_1}}-\bar{X_{t\_2}}\hat{\beta_{C}} \right)$ represents the differences between $t_{2}$ and $t_{1}$ scores attributable to the differences in characteristics of endowments and hence represents the covariate effect. $\hat{R^{Coeff}}$and $\hat{R^{Cov}}$are errors related to the estimation of coefficient and covariate effects.

**References**

| 1. | Firpo S Fortin NM, Lemieux T (2009) Unconditional quantile regressions. *Econometrica* 77(3):953-973. |
| --- | --- |
| 2. | DiNardo J, Fortin NM, Lemieux T (1996) Labor market institutions and the distribution of wages, 1973-1992: A Semiparametric Approach. *Econometrica* 64(5):1001-1044. |
| 3. | Firpo S, Fortin N, Lemieux T (2007): Decomposing wage distributions using recentered influence function regressions. Working Paper, University of British Columbia Mineo |
