# Supplementary material for: What Explains Cambodia’s Success in Reducing Child Stunting-2000-2014?
Source: PLoS One. 2016 Sep 20;11(9):e0162668. doi: 10.1371/journal.pone.0162668 (PMC5029902; doi:10.1371/journal.pone.0162668)
Supplement: S2 Appendix — (DOCX) [file pone.0162668.s002.docx]

# S2 Appendix: Unconditional RIF regression results for rural and urban Cambodia

Tables 1-12 below present the results of the Recentred Influence Function (RIF) quantile regression results separately for rural and urban areas of Cambodia, as well as nationwide. For each area, the RIF quantile regressions are estimated separately for each time period under analysis (2000-2014, 2000-2005, 2005-2010, and 2010-2014) and results for selected quantiles (10^th^, 25^th^, 50^th^, 75^th^ and 90^th^) are presented. The Ordinary Least Squares (OLS) estimates are also presented alongside for comparison. In these regressions, the dependent variable is the child HAZ score and the covariates are as listed in the tables. For any given quantile, the co-efficients of the RIF regression results can be interpreted as the “unconditional” effect of a unit change in the explanatory variable on child HAZ scores in standard deviation units. A coefficient of 1 for a continuous variable implies that a unit change in the variable leads to a one standard deviation change in the child HAZ score.

**Table 1: Unconditional RIF regression results for Cambodia 2000 - 2014**

|  | 2000 | | | | | | 2014 | | | | | |
| --- | --- | --- | --- | --- | --- | --- | --- | --- | --- | --- | --- | --- |
|  |  | Quantile regression - quantiles | | | | |  | Quantile regression - quantiles | | | | |
|  | OLS | 10^th^ | 25^th^ | 50^th^ | 75^th^ | 90^th^ | OLS | 10^th^ | 25^th^ | 50^th^ | 75^th^ | 90^th^ |
| Gender of child (0=male, 1=female) | 0.036 | -0.066 | -0.033 | 0.033 | 0.016 | 0.142 | 0.011 | 0.060 | -0.002 | 0.005 | -0.100 | -0.022 |
|  | (0.054) | (0.122) | (0.075) | (0.074) | (0.084) | (0.161) | (0.038) | (0.082) | (0.053) | (0.043) | (0.068) | (0.115) |
| Age of child (months) | -0.031*** | -0.019*** | -0.017*** | -0.025*** | -0.042*** | -0.054*** | -0.019*** | -0.006** | -0.008*** | -0.014*** | -0.026*** | -0.036*** |
|  | (0.002) | (0.004) | (0.003) | (0.003) | (0.004) | (0.005) | (0.001) | (0.002) | (0.002) | (0.002) | (0.003) | (0.004) |
| Birth in hospital | 0.067 | 0.217 | -0.078 | 0.125 | -0.047 | -0.160 | 0.133** | -0.013 | 0.127 | 0.062 | 0.133 | 0.120 |
|  | (0.108) | (0.163) | (0.142) | (0.159) | (0.210) | (0.264) | (0.060) | (0.121) | (0.103) | (0.079) | (0.107) | (0.132) |
| Breastfeeding within hour of birth | 0.027 | 0.054 | -0.003 | -0.020 | 0.042 | -0.142 | -0.051 | 0.010 | -0.022 | -0.073 | -0.097 | -0.062 |
|  | (0.088) | (0.193) | (0.123) | (0.113) | (0.142) | (0.197) | (0.041) | (0.074) | (0.055) | (0.060) | (0.068) | (0.086) |
| Prenatal visits | 0.092 | 0.086 | 0.029 | 0.021 | 0.138* | 0.135 | 0.029 | 0.341** | 0.129 | 0.103 | -0.107 | 0.040 |
|  | (0.061) | (0.134) | (0.093) | (0.063) | (0.079) | (0.182) | (0.070) | (0.151) | (0.085) | (0.104) | (0.121) | (0.180) |
| All recommended vaccinations | 0.133** | 0.282** | 0.225** | 0.059 | 0.016 | 0.352** | -0.114** | -0.063 | -0.123 | -0.157** | -0.229** | -0.065 |
|  | (0.061) | (0.140) | (0.090) | (0.080) | (0.111) | (0.149) | (0.045) | (0.079) | (0.078) | (0.063) | (0.101) | (0.128) |
| Dependency ratio | -0.102*** | -0.090 | -0.130*** | -0.097** | -0.050 | -0.161 | -0.045 | -0.083 | -0.145*** | -0.075* | -0.069 | 0.096 |
|  | (0.037) | (0.095) | (0.047) | (0.049) | (0.058) | (0.107) | (0.032) | (0.061) | (0.054) | (0.040) | (0.064) | (0.087) |
| BMI | 0.029*** | 0.020 | 0.031** | 0.018 | 0.043*** | 0.067** | 0.037*** | 0.031*** | 0.047*** | 0.036*** | 0.041*** | 0.024* |
|  | (0.011) | (0.024) | (0.013) | (0.015) | (0.017) | (0.033) | (0.006) | (0.011) | (0.010) | (0.009) | (0.011) | (0.013) |
| Mother currently working | 0.037 | 0.046 | 0.018 | 0.037 | 0.024 | 0.208 | -0.048 | -0.053 | -0.074 | -0.013 | -0.111 | 0.044 |
|  | (0.063) | (0.123) | (0.074) | (0.087) | (0.118) | (0.154) | (0.040) | (0.074) | (0.078) | (0.043) | (0.080) | (0.103) |
| Maternal education | 0.101 | -0.052 | 0.179** | 0.156* | 0.113 | 0.200 | -0.034 | 0.015 | -0.016 | -0.049 | -0.146 | -0.028 |
|  | (0.064) | (0.156) | (0.077) | (0.086) | (0.094) | (0.183) | (0.062) | (0.120) | (0.096) | (0.091) | (0.143) | (0.117) |
| Father’s education | 0.033 | 0.296 | 0.106 | -0.008 | -0.193 | -0.266 | 0.063 | 0.282* | 0.119 | 0.044 | 0.038 | 0.136 |
|  | (0.076) | (0.214) | (0.107) | (0.109) | (0.144) | (0.207) | (0.066) | (0.146) | (0.111) | (0.080) | (0.098) | (0.125) |
| Wealth index | 0.239*** | 0.235 | 0.245** | 0.238*** | 0.229** | 0.094 | 0.198*** | 0.094* | 0.154*** | 0.192*** | 0.211*** | 0.385*** |
|  | (0.060) | (0.148) | (0.097) | (0.084) | (0.116) | (0.139) | (0.030) | (0.049) | (0.041) | (0.041) | (0.056) | (0.074) |
| Improved water use | -0.108* | 0.001 | -0.104 | -0.095 | -0.174* | -0.157 | 0.095** | 0.029 | 0.098* | 0.125** | 0.077 | 0.108 |
|  | (0.058) | (0.105) | (0.087) | (0.082) | (0.101) | (0.168) | (0.040) | (0.057) | (0.058) | (0.063) | (0.075) | (0.120) |
| Improved sanitation | 0.005 | -0.201 | -0.162 | 0.160 | 0.107 | -0.065 | 0.103* | 0.035 | 0.065 | 0.182*** | 0.176* | 0.001 |
|  | (0.111) | (0.237) | (0.164) | (0.158) | (0.196) | (0.322) | (0.054) | (0.102) | (0.082) | (0.064) | (0.094) | (0.134) |
| Community sanitation | 0.079 | -0.149 | 0.154 | 0.284 | 0.225 | -0.060 | 0.161* | 0.217** | 0.198 | 0.095 | 0.316* | 0.064 |
|  | (0.164) | (0.415) | (0.263) | (0.231) | (0.266) | (0.541) | (0.084) | (0.111) | (0.132) | (0.135) | (0.165) | (0.259) |
| North West region | 0.229*** | 0.181 | 0.166** | 0.242*** | 0.332*** | 0.407** | 0.041 | -0.117 | 0.008 | 0.053 | 0.031 | 0.239** |
|  | (0.064) | (0.134) | (0.078) | (0.084) | (0.106) | (0.189) | (0.045) | (0.084) | (0.072) | (0.056) | (0.081) | (0.116) |
| North East region | -0.203 | -0.203 | -0.218 | -0.202** | -0.168 | -0.235 | 0.087 | 0.018 | 0.117 | 0.033 | 0.109 | 0.246* |
|  | (0.130) | (0.246) | (0.141) | (0.087) | (0.143) | (0.206) | (0.078) | (0.096) | (0.096) | (0.069) | (0.094) | (0.142) |
| South West region | 0.052 | -0.409 | -0.146 | 0.115 | 0.136 | 0.449 | 0.095 | 0.032 | 0.140 | 0.161* | 0.005 | -0.098 |
|  | (0.110) | (0.251) | (0.167) | (0.153) | (0.155) | (0.287) | (0.082) | (0.131) | (0.092) | (0.083) | (0.108) | (0.139) |
| Constant | -1.502*** | -3.779*** | -3.011*** | -1.587*** | -0.452 | 0.458 | -1.938*** | -4.050*** | -3.302*** | -2.109*** | -0.726** | 0.222 |
|  | (0.262) | (0.614) | (0.339) | (0.314) | (0.427) | (0.669) | (0.167) | (0.317) | (0.274) | (0.254) | (0.315) | (0.437) |
| N | 3390 | 3390 | 3390 | 3390 | 3390 | 3390 | 4240 | 4240 | 4240 | 4240 | 4240 | 4240 |
| R-sq | 0.125 | 0.025 | 0.045 | 0.087 | 0.109 | 0.064 | 0.126 | 0.026 | 0.059 | 0.089 | 0.108 | 0.079 |
| adj. R-sq | 0.120 | 0.020 | 0.040 | 0.082 | 0.104 | 0.059 | 0.123 | 0.022 | 0.055 | 0.085 | 0.104 | 0.075 |
| Figures in brackets are standard errors; asterisks show level of significance ***= significant at 1% level, **=significant at 5% level and *=significant at 10% level. | | | | | | | | | | | | |

**Table 2: Unconditional RIF regression results for rural Cambodia 2000 - 2014**

|  | 2000 | | | | | | 2014 | | | | | |
| --- | --- | --- | --- | --- | --- | --- | --- | --- | --- | --- | --- | --- |
|  |  | Quantile regression - quantiles | | | | |  | Quantile regression - quantiles | | | | |
|  | OLS | 10^th^ | 25^th^ | 50^th^ | 75^th^ | 90^th^ | OLS | 10^th^ | 25^th^ | 50^th^ | 75^th^ | 90^th^ |
| Gender of child (0=male, 1=female) | 0.045 | -0.084 | -0.055 | 0.025 | 0.061 | 0.172 | 0.020 | 0.051 | -0.009 | -0.008 | -0.036 | 0.101 |
|  | (0.058) | (0.098) | (0.079) | (0.063) | (0.096) | (0.192) | (0.044) | (0.061) | (0.067) | (0.054) | (0.084) | (0.125) |
| Age of child (months) | -0.033*** | -0.023*** | -0.019*** | -0.026*** | -0.045*** | -0.059*** | -0.019*** | -0.006** | -0.008*** | -0.013*** | -0.025*** | -0.040*** |
|  | (0.002) | (0.003) | (0.003) | (0.002) | (0.003) | (0.006) | (0.001) | (0.002) | (0.002) | (0.002) | (0.003) | (0.005) |
| Birth in hospital | 0.004 | 0.351* | -0.032 | 0.098 | -0.130 | -0.481* | 0.143** | 0.018 | 0.163 | 0.066 | 0.146 | 0.187 |
|  | (0.128) | (0.208) | (0.174) | (0.184) | (0.269) | (0.265) | (0.066) | (0.104) | (0.108) | (0.078) | (0.127) | (0.161) |
| Breastfeeding within hour of birth | -0.011 | -0.017 | -0.059 | -0.133 | 0.055 | -0.094 | -0.042 | 0.015 | -0.031 | -0.087 | -0.060 | -0.020 |
|  | (0.095) | (0.157) | (0.144) | (0.134) | (0.146) | (0.247) | (0.048) | (0.070) | (0.063) | (0.061) | (0.091) | (0.140) |
| Prenatal visits | 0.101 | 0.131 | 0.063 | 0.020 | 0.149 | 0.064 | 0.035 | 0.246 | 0.112 | 0.121 | -0.111 | 0.065 |
|  | (0.065) | (0.128) | (0.083) | (0.100) | (0.099) | (0.187) | (0.078) | (0.167) | (0.095) | (0.088) | (0.142) | (0.206) |
| All recommended vaccinations | 0.141** | 0.310** | 0.249*** | 0.109 | 0.105 | 0.266 | -0.123** | -0.066 | -0.145* | -0.172*** | -0.265*** | -0.119 |
|  | (0.066) | (0.134) | (0.089) | (0.087) | (0.109) | (0.226) | (0.052) | (0.081) | (0.079) | (0.061) | (0.095) | (0.156) |
| Dependency ratio | -0.087** | -0.069 | -0.119** | -0.078 | 0.002 | -0.149 | -0.031 | -0.053 | -0.155*** | -0.069 | -0.049 | 0.164* |
|  | (0.040) | (0.082) | (0.047) | (0.058) | (0.059) | (0.096) | (0.036) | (0.076) | (0.057) | (0.048) | (0.081) | (0.099) |
| BMI | 0.027** | 0.019 | 0.026 | 0.018 | 0.031 | 0.052 | 0.043*** | 0.038*** | 0.055*** | 0.042*** | 0.045*** | 0.031* |
|  | (0.012) | (0.026) | (0.017) | (0.014) | (0.023) | (0.035) | (0.007) | (0.010) | (0.009) | (0.010) | (0.016) | (0.017) |
| Mother currently working | 0.019 | 0.090 | 0.037 | 0.024 | -0.007 | 0.093 | -0.069 | -0.064 | -0.075 | -0.043 | -0.159 | -0.019 |
|  | (0.068) | (0.143) | (0.104) | (0.088) | (0.094) | (0.163) | (0.047) | (0.083) | (0.074) | (0.067) | (0.101) | (0.126) |
| Maternal education | 0.090 | -0.109 | 0.200* | 0.091 | 0.117 | 0.149 | -0.029 | 0.076 | -0.033 | -0.010 | -0.075 | 0.101 |
|  | (0.067) | (0.176) | (0.109) | (0.079) | (0.109) | (0.190) | (0.069) | (0.120) | (0.101) | (0.087) | (0.108) | (0.147) |
| Father’s education | 0.038 | 0.313 | 0.134 | 0.066 | -0.176 | -0.250 | 0.068 | 0.257* | 0.129 | 0.084 | -0.001 | 0.066 |
|  | (0.079) | (0.205) | (0.110) | (0.105) | (0.152) | (0.237) | (0.074) | (0.153) | (0.102) | (0.092) | (0.116) | (0.137) |
| Wealth index | 0.252*** | 0.353*** | 0.226** | 0.233*** | 0.245** | 0.068 | 0.167*** | 0.106 | 0.155*** | 0.128** | 0.151** | 0.296*** |
|  | (0.073) | (0.116) | (0.113) | (0.087) | (0.116) | (0.170) | (0.039) | (0.073) | (0.057) | (0.056) | (0.065) | (0.103) |
| Improved water use | -0.167*** | -0.020 | -0.134 | -0.130* | -0.276*** | -0.251 | 0.114** | 0.056 | 0.098 | 0.159** | 0.044 | 0.135 |
|  | (0.063) | (0.125) | (0.100) | (0.076) | (0.103) | (0.201) | (0.046) | (0.075) | (0.065) | (0.063) | (0.083) | (0.144) |
| Improved sanitation | 0.052 | -0.314 | -0.104 | 0.226 | 0.288 | 0.050 | 0.088 | 0.007 | 0.016 | 0.212** | 0.219** | -0.016 |
|  | (0.122) | (0.230) | (0.172) | (0.160) | (0.247) | (0.335) | (0.060) | (0.095) | (0.104) | (0.083) | (0.098) | (0.163) |
| Community sanitation | 0.110 | -0.043 | 0.178 | 0.189 | -0.013 | 0.074 | 0.214** | 0.228 | 0.294** | 0.069 | 0.283 | 0.117 |
|  | (0.201) | (0.339) | (0.300) | (0.279) | (0.330) | (0.591) | (0.098) | (0.151) | (0.119) | (0.150) | (0.182) | (0.263) |
| North West region | 0.274*** | 0.209 | 0.210** | 0.236*** | 0.331** | 0.439** | 0.015 | -0.092 | 0.016 | 0.033 | 0.007 | 0.140 |
|  | (0.069) | (0.131) | (0.087) | (0.073) | (0.131) | (0.177) | (0.052) | (0.073) | (0.064) | (0.070) | (0.087) | (0.132) |
| North East region | -0.112 | -0.166 | -0.120 | -0.072 | -0.064 | -0.293 | 0.095 | 0.017 | 0.131 | 0.043 | 0.137 | 0.345* |
|  | (0.149) | (0.259) | (0.138) | (0.109) | (0.156) | (0.307) | (0.090) | (0.131) | (0.106) | (0.088) | (0.122) | (0.196) |
| South West region | 0.132 | -0.189 | -0.130 | 0.125 | 0.162 | 0.628* | 0.082 | 0.062 | 0.196** | 0.188* | 0.017 | -0.307** |
|  | (0.133) | (0.331) | (0.200) | (0.146) | (0.207) | (0.381) | (0.098) | (0.142) | (0.095) | (0.104) | (0.141) | (0.143) |
| Constant | -1.369*** | -3.580*** | -2.915*** | -1.544*** | -0.193 | 1.046 | -2.088*** | -4.166*** | -3.482*** | -2.313*** | -0.897** | -0.045 |
|  | (0.287) | (0.620) | (0.453) | (0.267) | (0.445) | (0.813) | (0.193) | (0.327) | (0.319) | (0.251) | (0.412) | (0.454) |
| N | 2895 | 2895 | 2895 | 2895 | 2895 | 2895 | 3080 | 3080 | 3080 | 3080 | 3080 | 3080 |
| R-sq | 0.134 | 0.029 | 0.050 | 0.085 | 0.116 | 0.073 | 0.121 | 0.025 | 0.059 | 0.078 | 0.102 | 0.080 |
| adj. R-sq | 0.128 | 0.023 | 0.044 | 0.080 | 0.111 | 0.067 | 0.116 | 0.019 | 0.053 | 0.072 | 0.097 | 0.074 |
| Figures in brackets are standard errors; asterisks show level of significance ***= significant at 1% level, **=significant at 5% level and *=significant at 10% level. | | | | | | | | | | | | |

**Table 3: Unconditional RIF regression results for urban Cambodia 2000 - 2014**

|  | 2000 | | | | | | 2014 | | | | | |
| --- | --- | --- | --- | --- | --- | --- | --- | --- | --- | --- | --- | --- |
|  |  | Quantile regression - quantiles | | | | |  | Quantile regression - quantiles | | | | |
|  | OLS | 10^th^ | 25^th^ | 50^th^ | 75^th^ | 90^th^ | OLS | 10^th^ | 25^th^ | 50^th^ | 75^th^ | 90^th^ |
| Gender of child (0=male, 1=female) | 0.020 | 0.030 | 0.083 | -0.046 | -0.164 | 0.174 | -0.051 | 0.078 | 0.100 | -0.028 | -0.312* | -0.130 |
|  | (0.153) | (0.275) | (0.265) | (0.208) | (0.214) | (0.287) | (0.076) | (0.167) | (0.149) | (0.110) | (0.164) | (0.161) |
| Age of child (months) | -0.018*** | 0.000 | -0.004 | -0.019*** | -0.033*** | -0.024** | -0.016*** | -0.001 | -0.008 | -0.015*** | -0.022*** | -0.017*** |
|  | (0.004) | (0.008) | (0.006) | (0.006) | (0.007) | (0.010) | (0.003) | (0.006) | (0.005) | (0.004) | (0.004) | (0.006) |
| Birth in hospital | 0.073 | -0.126 | -0.147 | 0.323 | -0.021 | 0.248 | -0.078 | -0.504 | 0.069 | 0.083 | 0.116 | -0.114 |
|  | (0.220) | (0.319) | (0.389) | (0.307) | (0.354) | (0.437) | (0.234) | (0.509) | (0.454) | (0.281) | (0.354) | (0.257) |
| Breastfeeding within hour of birth | 0.229 | 0.416 | 0.339 | 0.498* | -0.124 | -0.315 | -0.075 | -0.068 | 0.110 | -0.144 | -0.255 | -0.283* |
|  | (0.250) | (0.434) | (0.428) | (0.295) | (0.335) | (0.464) | (0.079) | (0.161) | (0.137) | (0.120) | (0.188) | (0.168) |
| Prenatal visits | 0.099 | -0.128 | -0.183 | 0.118 | -0.019 | 0.201 | 0.102 | 0.392 | 0.171 | 0.146 | -0.355 | 0.152 |
|  | (0.179) | (0.298) | (0.320) | (0.275) | (0.268) | (0.353) | (0.195) | (0.412) | (0.293) | (0.202) | (0.358) | (0.252) |
| All recommended vaccinations | 0.168 | 0.269 | -0.025 | -0.039 | 0.030 | 0.634* | -0.083 | -0.185 | 0.087 | -0.092 | -0.122 | -0.139 |
|  | (0.164) | (0.302) | (0.416) | (0.281) | (0.232) | (0.364) | (0.100) | (0.143) | (0.163) | (0.152) | (0.233) | (0.200) |
| Dependency ratio | -0.150 | -0.144 | -0.183 | -0.023 | -0.036 | -0.094 | -0.175** | -0.176 | -0.237* | -0.229* | -0.084 | -0.102 |
|  | (0.103) | (0.216) | (0.228) | (0.118) | (0.138) | (0.202) | (0.074) | (0.144) | (0.137) | (0.117) | (0.154) | (0.131) |
| BMI | 0.034 | 0.013 | 0.039 | 0.032 | 0.075 | 0.088 | 0.006 | 0.007 | -0.004 | 0.002 | 0.008 | 0.000 |
|  | (0.029) | (0.058) | (0.048) | (0.045) | (0.051) | (0.081) | (0.011) | (0.024) | (0.017) | (0.014) | (0.027) | (0.025) |
| Mother currently working | 0.162 | -0.114 | -0.073 | 0.017 | 0.226 | 0.622 | 0.098 | 0.005 | 0.018 | 0.126 | 0.150 | 0.234 |
|  | (0.162) | (0.308) | (0.340) | (0.303) | (0.239) | (0.390) | (0.081) | (0.173) | (0.122) | (0.094) | (0.184) | (0.200) |
| Maternal education | 0.189 | 0.184 | -0.088 | 0.371 | 0.303 | 0.589 | 0.050 | -0.068 | 0.089 | 0.164 | -0.041 | 0.008 |
|  | (0.202) | (0.456) | (0.401) | (0.255) | (0.252) | (0.417) | (0.193) | (0.481) | (0.358) | (0.287) | (0.425) | (0.172) |
| Father’s education | 0.110 | 0.296 | 0.137 | 0.079 | 0.075 | 0.145 | -0.181 | -0.303 | -0.247 | -0.189 | -0.259 | 0.013 |
|  | (0.254) | (0.638) | (0.439) | (0.385) | (0.266) | (0.476) | (0.214) | (0.356) | (0.339) | (0.341) | (0.499) | (0.158) |
| Wealth index | 0.184 | -0.009 | 0.496* | 0.136 | 0.279* | -0.056 | 0.291*** | 0.189** | 0.340*** | 0.304*** | 0.269** | 0.240** |
|  | (0.126) | (0.243) | (0.296) | (0.198) | (0.166) | (0.292) | (0.048) | (0.091) | (0.088) | (0.070) | (0.136) | (0.115) |
| Improved water use | 0.214 | 0.244 | 0.273 | 0.148 | 0.019 | 0.381 | -0.026 | 0.027 | 0.116 | 0.035 | 0.171 | -0.165 |
|  | (0.162) | (0.332) | (0.313) | (0.274) | (0.194) | (0.352) | (0.088) | (0.179) | (0.138) | (0.148) | (0.164) | (0.217) |
| Improved sanitation | -0.201 | 0.282 | -0.470 | -0.292 | -0.213 | -0.333 | 0.494*** | 0.928** | 0.719*** | 0.040 | 0.412* | 0.266 |
|  | (0.272) | (0.655) | (0.463) | (0.374) | (0.442) | (0.481) | (0.168) | (0.383) | (0.276) | (0.201) | (0.247) | (0.186) |
| Community sanitation | 0.254 | -0.273 | 0.509 | 1.037** | 0.099 | -0.139 | -0.134 | -0.029 | -0.613 | -0.121 | 0.171 | -0.133 |
|  | (0.352) | (0.784) | (0.638) | (0.434) | (0.496) | (0.621) | (0.261) | (0.469) | (0.385) | (0.347) | (0.413) | (0.439) |
| North West region | 0.089 | -0.036 | 0.143 | 0.207 | 0.250 | 0.374 | 0.173* | 0.009 | 0.090 | 0.170 | 0.159 | 0.101 |
|  | (0.217) | (0.316) | (0.403) | (0.327) | (0.313) | (0.481) | (0.103) | (0.207) | (0.132) | (0.136) | (0.217) | (0.200) |
| North East region | -0.551* | -0.498 | -0.617 | -0.614 | -0.432 | -0.209 | 0.089 | 0.284 | 0.236 | 0.124 | -0.076 | -0.088 |
|  | (0.316) | (0.462) | (0.534) | (0.381) | (0.362) | (0.426) | (0.177) | (0.210) | (0.187) | (0.194) | (0.178) | (0.187) |
| South West region | -0.057 | -0.922** | -0.087 | 0.052 | 0.112 | 0.138 | 0.167 | 0.044 | 0.092 | -0.090 | 0.215 | 0.313 |
|  | (0.261) | (0.464) | (0.557) | (0.495) | (0.360) | (0.438) | (0.153) | (0.272) | (0.226) | (0.170) | (0.229) | (0.261) |
| Constant | -2.317*** | -4.272*** | -3.367*** | -2.811*** | -1.518 | -2.278 | -1.213*** | -3.113*** | -2.365*** | -1.137** | 0.015 | 0.778 |
|  | (0.719) | (1.222) | (1.192) | (1.004) | (1.326) | (1.510) | (0.463) | (0.670) | (0.630) | (0.545) | (0.930) | (0.729) |
| N | 495 | 495 | 495 | 495 | 495 | 495 | 1160 | 1160 | 1160 | 1160 | 1160 | 1160 |
| R-sq | 0.109 | 0.035 | 0.067 | 0.121 | 0.113 | 0.059 | 0.129 | 0.052 | 0.085 | 0.104 | 0.086 | 0.064 |
| adj. R-sq | 0.076 | -0.002 | 0.032 | 0.088 | 0.079 | 0.024 | 0.115 | 0.037 | 0.070 | 0.089 | 0.072 | 0.049 |
| Figures in brackets are standard errors; asterisks show level of significance ***= significant at 1% level, **=significant at 5% level and *=significant at 10% level. | | | | | | | | | | | | |

**Table 4: Unconditional RIF regression results for Cambodia 2000 - 2005**

|  | 2000 | | | | | | 2005 | | | | | |
| --- | --- | --- | --- | --- | --- | --- | --- | --- | --- | --- | --- | --- |
|  |  | Quantile regression - quantiles | | | | |  | Quantile regression - quantiles | | | | |
|  | OLS | 10^th^ | 25^th^ | 50^th^ | 75^th^ | 90^th^ | OLS | 10^th^ | 25^th^ | 50^th^ | 75^th^ | 90^th^ |
| Gender of child (0=male, 1=female) | 0.036 | -0.066 | -0.033 | 0.033 | 0.016 | 0.142 | 0.160*** | 0.234** | 0.242*** | 0.130** | 0.108* | 0.204* |
|  | (0.054) | (0.114) | (0.075) | (0.075) | (0.091) | (0.155) | (0.041) | (0.100) | (0.063) | (0.054) | (0.064) | (0.121) |
| Age of child (months) | -0.031*** | -0.019*** | -0.017*** | -0.025*** | -0.042*** | -0.054*** | -0.025*** | -0.012*** | -0.018*** | -0.022*** | -0.032*** | -0.041*** |
|  | (0.002) | (0.003) | (0.002) | (0.003) | (0.003) | (0.007) | (0.001) | (0.003) | (0.002) | (0.002) | (0.003) | (0.004) |
| Birth in hospital | 0.067 | 0.217 | -0.078 | 0.125 | -0.047 | -0.160 | -0.084 | -0.160 | -0.297*** | -0.060 | 0.104 | 0.079 |
|  | (0.108) | (0.183) | (0.150) | (0.145) | (0.229) | (0.271) | (0.059) | (0.110) | (0.112) | (0.094) | (0.105) | (0.159) |
| Breastfeeding within hour of birth | 0.027 | 0.054 | -0.003 | -0.020 | 0.042 | -0.142 | 0.046 | 0.069 | 0.100 | 0.030 | 0.070 | 0.000 |
|  | (0.088) | (0.195) | (0.109) | (0.100) | (0.154) | (0.239) | (0.043) | (0.100) | (0.083) | (0.050) | (0.074) | (0.120) |
| Prenatal visits | 0.092 | 0.086 | 0.029 | 0.021 | 0.138 | 0.135 | 0.195*** | 0.166 | 0.213*** | 0.106 | 0.187*** | 0.380*** |
|  | (0.061) | (0.144) | (0.083) | (0.091) | (0.098) | (0.168) | (0.045) | (0.108) | (0.075) | (0.065) | (0.064) | (0.117) |
| All recommended vaccinations | 0.133** | 0.282** | 0.225** | 0.059 | 0.016 | 0.352* | -0.085* | 0.034 | 0.058 | -0.050 | -0.179** | -0.257** |
|  | (0.061) | (0.121) | (0.090) | (0.069) | (0.114) | (0.186) | (0.044) | (0.106) | (0.094) | (0.080) | (0.082) | (0.119) |
| Dependency ratio | -0.102*** | -0.090 | -0.130*** | -0.097** | -0.050 | -0.161 | -0.053* | -0.077 | -0.064 | -0.045 | -0.021 | -0.074 |
|  | (0.037) | (0.068) | (0.047) | (0.047) | (0.054) | (0.111) | (0.027) | (0.062) | (0.042) | (0.044) | (0.047) | (0.053) |
| BMI | 0.029*** | 0.020 | 0.031* | 0.018 | 0.043*** | 0.067** | 0.027*** | 0.035** | 0.028** | 0.015 | 0.013 | 0.021 |
|  | (0.011) | (0.022) | (0.016) | (0.013) | (0.014) | (0.030) | (0.008) | (0.016) | (0.011) | (0.011) | (0.012) | (0.022) |
| Mother currently working | 0.037 | 0.046 | 0.018 | 0.037 | 0.024 | 0.208 | -0.065 | -0.012 | -0.068 | -0.025 | -0.082 | -0.230* |
|  | (0.063) | (0.117) | (0.093) | (0.083) | (0.112) | (0.184) | (0.043) | (0.094) | (0.063) | (0.062) | (0.065) | (0.133) |
| Maternal education | 0.101 | -0.052 | 0.179* | 0.156* | 0.113 | 0.200 | 0.063 | 0.235** | 0.069 | 0.017 | -0.059 | -0.001 |
|  | (0.064) | (0.132) | (0.098) | (0.081) | (0.093) | (0.177) | (0.053) | (0.112) | (0.078) | (0.083) | (0.072) | (0.134) |
| Father’s education | 0.033 | 0.296 | 0.106 | -0.008 | -0.193 | -0.266 | 0.161** | 0.224 | 0.334*** | 0.115 | 0.059 | -0.091 |
|  | (0.076) | (0.205) | (0.131) | (0.093) | (0.121) | (0.215) | (0.063) | (0.144) | (0.115) | (0.079) | (0.108) | (0.161) |
| Wealth index | 0.170*** | 0.167* | 0.173*** | 0.169*** | 0.162** | 0.067 | 0.176*** | 0.093 | 0.206*** | 0.174*** | 0.165*** | 0.267** |
|  | (0.043) | (0.096) | (0.063) | (0.060) | (0.074) | (0.120) | (0.030) | (0.059) | (0.047) | (0.037) | (0.054) | (0.106) |
| Improved water use | -0.108* | 0.001 | -0.104 | -0.095 | -0.174 | -0.157 | 0.028 | -0.012 | 0.042 | 0.046 | -0.002 | -0.000 |
|  | (0.058) | (0.123) | (0.083) | (0.069) | (0.106) | (0.172) | (0.044) | (0.111) | (0.085) | (0.065) | (0.069) | (0.124) |
| Improved sanitation | 0.005 | -0.201 | -0.162 | 0.160 | 0.107 | -0.065 | -0.064 | -0.057 | -0.099 | -0.046 | 0.100 | -0.130 |
|  | (0.111) | (0.198) | (0.161) | (0.130) | (0.195) | (0.284) | (0.081) | (0.183) | (0.135) | (0.124) | (0.142) | (0.243) |
| Community sanitation | 0.079 | -0.149 | 0.154 | 0.284 | 0.225 | -0.060 | 0.467*** | 0.562** | 0.533*** | 0.514*** | 0.311 | 0.567** |
|  | (0.164) | (0.398) | (0.256) | (0.195) | (0.278) | (0.501) | (0.112) | (0.241) | (0.177) | (0.153) | (0.216) | (0.273) |
| North West region | 0.229*** | 0.181 | 0.166** | 0.242*** | 0.332*** | 0.407** | -0.244*** | -0.159 | -0.229** | -0.290*** | -0.269*** | -0.182* |
|  | (0.064) | (0.148) | (0.083) | (0.078) | (0.108) | (0.205) | (0.049) | (0.098) | (0.103) | (0.077) | (0.073) | (0.102) |
| North East region | -0.203 | -0.203 | -0.218* | -0.202* | -0.168 | -0.235 | -0.269*** | -0.412*** | -0.121 | -0.285*** | -0.205** | -0.179 |
|  | (0.130) | (0.226) | (0.117) | (0.118) | (0.156) | (0.269) | (0.093) | (0.133) | (0.137) | (0.102) | (0.094) | (0.143) |
| South West region | 0.052 | -0.409 | -0.146 | 0.115 | 0.136 | 0.449 | 0.107 | 0.122 | 0.107 | 0.012 | 0.199 | 0.022 |
|  | (0.110) | (0.252) | (0.165) | (0.135) | (0.147) | (0.308) | (0.081) | (0.123) | (0.098) | (0.097) | (0.126) | (0.198) |
| Constant | -1.628*** | -3.903*** | -3.140*** | -1.713*** | -0.573 | 0.408 | -1.910*** | -4.328*** | -3.225*** | -1.674*** | -0.441 | 0.623 |
|  | (0.253) | (0.575) | (0.397) | (0.316) | (0.362) | (0.601) | (0.174) | (0.325) | (0.259) | (0.239) | (0.270) | (0.442) |
| N | 3390 | 3390 | 3390 | 3390 | 3390 | 3390 | 3407 | 3407 | 3407 | 3407 | 3407 | 3407 |
| R-sq | 0.125 | 0.025 | 0.045 | 0.087 | 0.109 | 0.064 | 0.190 | 0.046 | 0.091 | 0.126 | 0.159 | 0.107 |
| adj. R-sq | 0.120 | 0.020 | 0.040 | 0.082 | 0.104 | 0.059 | 0.185 | 0.041 | 0.086 | 0.122 | 0.154 | 0.102 |
| Figures in brackets are standard errors; asterisks show level of significance ***= significant at 1% level, **=significant at 5% level and *=significant at 10% level. | | | | | | | | | | | | |

**Table 5: Unconditional RIF regression results for rural Cambodia 2000 - 2005**

|  | 2000 | | | | | | 2005 | | | | | |
| --- | --- | --- | --- | --- | --- | --- | --- | --- | --- | --- | --- | --- |
|  |  | Quantile regression - quantiles | | | | |  | Quantile regression - quantiles | | | | |
|  | OLS | 10^th^ | 25^th^ | 50^th^ | 75^th^ | 90^th^ | OLS | 10^th^ | 25^th^ | 50^th^ | 75^th^ | 90^th^ |
| Gender of child (0=male, 1=female) | 0.045 | -0.084 | -0.055 | 0.025 | 0.061 | 0.172 | 0.160*** | 0.259*** | 0.279*** | 0.136** | 0.075 | 0.109 |
|  | (0.058) | (0.108) | (0.082) | (0.075) | (0.106) | (0.182) | (0.046) | (0.088) | (0.074) | (0.057) | (0.079) | (0.105) |
| Age of child (months) | -0.033*** | -0.023*** | -0.019*** | -0.026*** | -0.045*** | -0.059*** | -0.025*** | -0.012*** | -0.018*** | -0.022*** | -0.032*** | -0.046*** |
|  | (0.002) | (0.004) | (0.003) | (0.003) | (0.004) | (0.007) | (0.001) | (0.003) | (0.002) | (0.002) | (0.003) | (0.005) |
| Birth in hospital | 0.004 | 0.351** | -0.032 | 0.098 | -0.130 | -0.481 | -0.094 | -0.155 | -0.287** | -0.046 | 0.031 | 0.004 |
|  | (0.128) | (0.170) | (0.184) | (0.190) | (0.229) | (0.365) | (0.067) | (0.127) | (0.117) | (0.114) | (0.118) | (0.175) |
| Breastfeeding within hour of birth | -0.011 | -0.017 | -0.059 | -0.133 | 0.055 | -0.094 | 0.029 | 0.032 | 0.070 | -0.031 | 0.056 | 0.077 |
|  | (0.095) | (0.170) | (0.098) | (0.125) | (0.160) | (0.294) | (0.048) | (0.104) | (0.071) | (0.063) | (0.078) | (0.107) |
| Prenatal visits | 0.101 | 0.131 | 0.063 | 0.020 | 0.149 | 0.064 | 0.193*** | 0.129 | 0.235*** | 0.142** | 0.201** | 0.364*** |
|  | (0.065) | (0.166) | (0.083) | (0.086) | (0.095) | (0.193) | (0.049) | (0.118) | (0.088) | (0.072) | (0.083) | (0.112) |
| All recommended vaccinations | 0.141** | 0.310** | 0.249*** | 0.109 | 0.105 | 0.266* | -0.104** | 0.026 | 0.010 | -0.107 | -0.192** | -0.312** |
|  | (0.066) | (0.147) | (0.092) | (0.084) | (0.136) | (0.137) | (0.049) | (0.100) | (0.091) | (0.069) | (0.081) | (0.129) |
| Dependency ratio | -0.087** | -0.069 | -0.119*** | -0.078* | 0.002 | -0.149 | -0.042 | -0.040 | -0.013 | -0.032 | -0.028 | -0.091 |
|  | (0.040) | (0.097) | (0.045) | (0.041) | (0.077) | (0.101) | (0.030) | (0.073) | (0.051) | (0.039) | (0.045) | (0.065) |
| BMI | 0.027** | 0.019 | 0.026 | 0.018 | 0.031 | 0.052** | 0.032*** | 0.038** | 0.032* | 0.018 | 0.017 | 0.033* |
|  | (0.012) | (0.029) | (0.018) | (0.014) | (0.021) | (0.025) | (0.009) | (0.019) | (0.017) | (0.012) | (0.013) | (0.019) |
| Mother currently working | 0.019 | 0.090 | 0.037 | 0.024 | -0.007 | 0.093 | -0.078 | 0.025 | -0.059 | -0.017 | -0.111 | -0.255** |
|  | (0.068) | (0.153) | (0.093) | (0.094) | (0.104) | (0.219) | (0.048) | (0.093) | (0.070) | (0.062) | (0.070) | (0.106) |
| Maternal education | 0.090 | -0.109 | 0.200** | 0.091 | 0.117 | 0.149 | 0.053 | 0.235** | 0.040 | -0.004 | -0.034 | 0.068 |
|  | (0.067) | (0.138) | (0.083) | (0.085) | (0.111) | (0.205) | (0.059) | (0.110) | (0.101) | (0.090) | (0.096) | (0.102) |
| Father’s education | 0.038 | 0.313 | 0.134 | 0.066 | -0.176 | -0.250 | 0.218*** | 0.200 | 0.423*** | 0.182** | 0.127 | -0.072 |
|  | (0.079) | (0.226) | (0.107) | (0.133) | (0.147) | (0.245) | (0.070) | (0.160) | (0.149) | (0.091) | (0.108) | (0.177) |
| Wealth index | 0.178*** | 0.250*** | 0.160** | 0.165*** | 0.173* | 0.048 | 0.134*** | 0.105 | 0.207*** | 0.135** | 0.099 | 0.167 |
|  | (0.052) | (0.080) | (0.073) | (0.059) | (0.096) | (0.142) | (0.036) | (0.079) | (0.060) | (0.054) | (0.061) | (0.110) |
| Improved water use | -0.167*** | -0.020 | -0.134 | -0.130 | -0.276*** | -0.251 | 0.035 | 0.017 | 0.025 | 0.050 | 0.003 | 0.045 |
|  | (0.063) | (0.141) | (0.090) | (0.087) | (0.097) | (0.179) | (0.049) | (0.096) | (0.091) | (0.070) | (0.082) | (0.135) |
| Improved sanitation | 0.052 | -0.314 | -0.104 | 0.226 | 0.288 | 0.050 | -0.058 | -0.114 | -0.135 | -0.036 | 0.019 | -0.101 |
|  | (0.122) | (0.225) | (0.157) | (0.157) | (0.203) | (0.361) | (0.092) | (0.159) | (0.174) | (0.134) | (0.173) | (0.242) |
| Community sanitation | 0.110 | -0.043 | 0.178 | 0.189 | -0.013 | 0.074 | 0.450*** | 0.605*** | 0.541*** | 0.504** | 0.363 | 0.389 |
|  | (0.201) | (0.432) | (0.278) | (0.224) | (0.375) | (0.555) | (0.134) | (0.197) | (0.210) | (0.206) | (0.301) | (0.387) |
| North West region | 0.274*** | 0.209* | 0.210** | 0.236** | 0.331*** | 0.439** | -0.254*** | -0.165 | -0.193** | -0.293*** | -0.284*** | -0.261** |
|  | (0.069) | (0.111) | (0.091) | (0.101) | (0.114) | (0.189) | (0.054) | (0.152) | (0.097) | (0.056) | (0.077) | (0.133) |
| North East region | -0.112 | -0.166 | -0.120 | -0.072 | -0.064 | -0.293 | -0.341*** | -0.528*** | -0.160 | -0.320*** | -0.237** | -0.199 |
|  | (0.149) | (0.182) | (0.138) | (0.105) | (0.167) | (0.275) | (0.112) | (0.187) | (0.135) | (0.099) | (0.119) | (0.161) |
| South West region | 0.132 | -0.189 | -0.130 | 0.125 | 0.162 | 0.628 | 0.168* | 0.213 | 0.184 | 0.054 | 0.276** | 0.050 |
|  | (0.133) | (0.241) | (0.159) | (0.172) | (0.198) | (0.409) | (0.095) | (0.148) | (0.136) | (0.127) | (0.136) | (0.198) |
| Constant | -1.502*** | -3.766*** | -3.035*** | -1.667*** | -0.322 | 1.010* | -2.013*** | -4.402*** | -3.411*** | -1.756*** | -0.515* | 0.581 |
|  | (0.275) | (0.700) | (0.413) | (0.364) | (0.474) | (0.609) | (0.201) | (0.422) | (0.393) | (0.276) | (0.290) | (0.444) |
| N | 2895 | 2895 | 2895 | 2895 | 2895 | 2895 | 2722 | 2722 | 2722 | 2722 | 2722 | 2722 |
| R-sq | 0.134 | 0.029 | 0.050 | 0.085 | 0.116 | 0.073 | 0.174 | 0.044 | 0.081 | 0.110 | 0.146 | 0.119 |
| adj. R-sq | 0.128 | 0.023 | 0.044 | 0.080 | 0.111 | 0.067 | 0.169 | 0.038 | 0.075 | 0.104 | 0.140 | 0.113 |
| Figures in brackets are standard errors; asterisks show level of significance ***= significant at 1% level, **=significant at 5% level and *=significant at 10% level. | | | | | | | | | | | | |

**Table 6: Unconditional RIF regression results for urban Cambodia 2000 - 2005**

|  | 2000 | | | | | | 2005 | | | | | |
| --- | --- | --- | --- | --- | --- | --- | --- | --- | --- | --- | --- | --- |
|  |  | Quantile regression - quantiles | | | | |  | Quantile regression - quantiles | | | | |
|  | OLS | 10^th^ | 25^th^ | 50^th^ | 75^th^ | 90^th^ | OLS | 10^th^ | 25^th^ | 50^th^ | 75^th^ | 90^th^ |
| Gender of child (0=male, 1=female) | 0.020 | 0.030 | 0.083 | -0.046 | -0.164 | 0.174 | 0.126 | -0.128 | 0.034 | 0.052 | 0.374** | 0.165 |
|  | (0.153) | (0.259) | (0.268) | (0.193) | (0.190) | (0.354) | (0.093) | (0.221) | (0.136) | (0.159) | (0.162) | (0.251) |
| Age of child (months) | -0.018*** | 0.000 | -0.004 | -0.019*** | -0.033*** | -0.024* | -0.022*** | -0.015** | -0.018*** | -0.017*** | -0.025*** | -0.022** |
|  | (0.004) | (0.007) | (0.007) | (0.007) | (0.008) | (0.012) | (0.003) | (0.006) | (0.005) | (0.004) | (0.006) | (0.009) |
| Birth in hospital | 0.073 | -0.126 | -0.147 | 0.323 | -0.021 | 0.248 | -0.026 | -0.177 | -0.229 | -0.035 | 0.350* | 0.011 |
|  | (0.220) | (0.420) | (0.333) | (0.281) | (0.338) | (0.404) | (0.120) | (0.323) | (0.274) | (0.202) | (0.180) | (0.212) |
| Breastfeeding within hour of birth | 0.229 | 0.416 | 0.339 | 0.498 | -0.124 | -0.315 | 0.219** | 0.261 | 0.116 | 0.129 | 0.308 | -0.050 |
|  | (0.250) | (0.462) | (0.328) | (0.326) | (0.400) | (0.452) | (0.096) | (0.189) | (0.147) | (0.134) | (0.204) | (0.285) |
| Prenatal visits | 0.099 | -0.128 | -0.183 | 0.118 | -0.019 | 0.201 | 0.190* | 0.207 | 0.219 | 0.115 | 0.228 | 0.472* |
|  | (0.179) | (0.319) | (0.382) | (0.258) | (0.249) | (0.340) | (0.112) | (0.297) | (0.199) | (0.153) | (0.192) | (0.274) |
| All recommended vaccinations | 0.168 | 0.269 | -0.025 | -0.039 | 0.030 | 0.634 | 0.109 | 0.120 | 0.369** | 0.396** | -0.067 | -0.272 |
|  | (0.164) | (0.316) | (0.297) | (0.273) | (0.251) | (0.408) | (0.100) | (0.300) | (0.152) | (0.160) | (0.161) | (0.295) |
| Dependency ratio | -0.150 | -0.144 | -0.183 | -0.023 | -0.036 | -0.094 | -0.074 | -0.345* | -0.190 | -0.048 | -0.081 | 0.093 |
|  | (0.103) | (0.203) | (0.182) | (0.125) | (0.115) | (0.247) | (0.057) | (0.203) | (0.120) | (0.080) | (0.105) | (0.144) |
| BMI | 0.034 | 0.013 | 0.039 | 0.032 | 0.075** | 0.088 | 0.017 | 0.031 | 0.030 | 0.008 | 0.023 | -0.010 |
|  | (0.029) | (0.065) | (0.043) | (0.041) | (0.037) | (0.080) | (0.015) | (0.030) | (0.025) | (0.026) | (0.030) | (0.042) |
| Mother currently working | 0.162 | -0.114 | -0.073 | 0.017 | 0.226 | 0.622 | -0.038 | -0.177 | -0.113 | -0.234 | 0.022 | 0.444 |
|  | (0.162) | (0.310) | (0.274) | (0.236) | (0.239) | (0.392) | (0.096) | (0.198) | (0.173) | (0.163) | (0.220) | (0.282) |
| Maternal education | 0.189 | 0.184 | -0.088 | 0.371 | 0.303 | 0.589* | 0.042 | -0.278 | 0.188 | -0.032 | -0.316 | 0.205 |
|  | (0.202) | (0.441) | (0.347) | (0.307) | (0.258) | (0.341) | (0.126) | (0.292) | (0.232) | (0.180) | (0.198) | (0.247) |
| Father’s education | 0.110 | 0.296 | 0.137 | 0.079 | 0.075 | 0.145 | -0.339** | 0.311 | -0.204 | -0.376 | -0.511* | -0.853 |
|  | (0.254) | (0.570) | (0.416) | (0.320) | (0.322) | (0.464) | (0.159) | (0.361) | (0.273) | (0.240) | (0.290) | (0.612) |
| Wealth index | 0.130 | -0.006 | 0.351** | 0.097 | 0.198 | -0.040 | 0.288*** | 0.221** | 0.333*** | 0.316*** | 0.225* | 0.236 |
|  | (0.089) | (0.185) | (0.154) | (0.132) | (0.143) | (0.166) | (0.053) | (0.099) | (0.071) | (0.113) | (0.127) | (0.145) |
| Improved water use | 0.214 | 0.244 | 0.273 | 0.148 | 0.019 | 0.381 | -0.130 | -0.073 | 0.175 | -0.130 | -0.319 | -0.617** |
|  | (0.162) | (0.344) | (0.246) | (0.271) | (0.264) | (0.273) | (0.111) | (0.320) | (0.184) | (0.167) | (0.197) | (0.266) |
| Improved sanitation | -0.201 | 0.282 | -0.470 | -0.292 | -0.213 | -0.333 | 0.078 | 0.044 | -0.048 | -0.026 | 0.389 | 0.182 |
|  | (0.272) | (0.598) | (0.465) | (0.451) | (0.444) | (0.491) | (0.175) | (0.388) | (0.328) | (0.289) | (0.428) | (0.344) |
| Community sanitation | 0.254 | -0.273 | 0.509 | 1.037** | 0.099 | -0.139 | 0.384* | 0.382 | 0.384 | 0.407 | 0.288 | 0.514 |
|  | (0.352) | (0.886) | (0.614) | (0.485) | (0.499) | (0.810) | (0.221) | (0.451) | (0.408) | (0.354) | (0.479) | (0.464) |
| North West region | 0.089 | -0.036 | 0.143 | 0.207 | 0.250 | 0.374 | -0.235* | -0.013 | 0.037 | -0.341 | -0.443* | -0.454 |
|  | (0.217) | (0.365) | (0.387) | (0.364) | (0.371) | (0.440) | (0.124) | (0.288) | (0.190) | (0.207) | (0.255) | (0.372) |
| North East region | -0.551* | -0.498 | -0.617 | -0.614* | -0.432 | -0.209 | -0.028 | 0.131 | 0.410* | -0.129 | -0.198 | -0.343 |
|  | (0.316) | (0.477) | (0.484) | (0.337) | (0.414) | (0.482) | (0.175) | (0.329) | (0.248) | (0.219) | (0.233) | (0.351) |
| South West region | -0.057 | -0.922* | -0.087 | 0.052 | 0.112 | 0.138 | -0.003 | -0.107 | 0.328 | -0.034 | -0.328 | -0.199 |
|  | (0.261) | (0.533) | (0.414) | (0.432) | (0.409) | (0.450) | (0.155) | (0.433) | (0.282) | (0.255) | (0.271) | (0.355) |
| Constant | -2.414*** | -4.267*** | -3.629*** | -2.883*** | -1.666* | -2.248 | -1.610*** | -3.595*** | -3.315*** | -1.428** | -0.433 | 1.064 |
|  | (0.712) | (1.426) | (1.131) | (1.070) | (1.008) | (1.654) | (0.352) | (0.793) | (0.674) | (0.616) | (0.834) | (1.136) |
| N | 495 | 495 | 495 | 495 | 495 | 495 | 685 | 685 | 685 | 685 | 685 | 685 |
| R-sq | 0.109 | 0.035 | 0.067 | 0.121 | 0.113 | 0.059 | 0.287 | 0.096 | 0.187 | 0.206 | 0.197 | 0.099 |
| adj. R-sq | 0.076 | -0.002 | 0.032 | 0.088 | 0.079 | 0.024 | 0.268 | 0.072 | 0.165 | 0.185 | 0.175 | 0.075 |
| Figures in brackets are standard errors; asterisks show level of significance ***= significant at 1% level, **=significant at 5% level and *=significant at 10% level. | | | | | | | | | | | | |

**Table 7: Unconditional RIF regression results for Cambodia 2005 - 2010**

|  | 2005 | | | | | | 2010 | | | | | |
| --- | --- | --- | --- | --- | --- | --- | --- | --- | --- | --- | --- | --- |
|  |  | Quantile regression - quantiles | | | | |  | Quantile regression - quantiles | | | | |
|  | OLS | 10^th^ | 25^th^ | 50^th^ | 75^th^ | 90^th^ | OLS | 10^th^ | 25^th^ | 50^th^ | 75^th^ | 90^th^ |
| Gender of child (0=male, 1=female) | 0.160*** | 0.234** | 0.242*** | 0.130** | 0.108 | 0.204* | -0.013 | -0.018 | 0.045 | 0.034 | -0.032 | -0.085 |
|  | (0.041) | (0.097) | (0.065) | (0.051) | (0.068) | (0.111) | (0.042) | (0.104) | (0.062) | (0.055) | (0.068) | (0.098) |
| Age of child (months) | -0.025*** | -0.012*** | -0.018*** | -0.022*** | -0.032*** | -0.041*** | -0.021*** | -0.016*** | -0.011*** | -0.016*** | -0.027*** | -0.038*** |
|  | (0.001) | (0.003) | (0.002) | (0.002) | (0.003) | (0.005) | (0.001) | (0.004) | (0.002) | (0.002) | (0.003) | (0.004) |
| Birth in hospital | -0.084 | -0.160 | -0.297** | -0.060 | 0.104 | 0.079 | 0.107** | -0.001 | 0.163** | 0.136* | 0.203*** | 0.114 |
|  | (0.059) | (0.109) | (0.119) | (0.092) | (0.125) | (0.182) | (0.049) | (0.114) | (0.065) | (0.071) | (0.076) | (0.136) |
| Breastfeeding within hour of birth | 0.046 | 0.069 | 0.100 | 0.030 | 0.070 | 0.000 | 0.129*** | 0.217** | 0.128* | 0.076 | 0.048 | 0.135 |
|  | (0.043) | (0.100) | (0.078) | (0.066) | (0.072) | (0.133) | (0.044) | (0.110) | (0.071) | (0.063) | (0.053) | (0.116) |
| Prenatal visits | 0.195*** | 0.166 | 0.213*** | 0.106* | 0.187** | 0.380*** | 0.088 | 0.375** | 0.145* | 0.103 | 0.035 | 0.048 |
|  | (0.045) | (0.104) | (0.070) | (0.062) | (0.083) | (0.129) | (0.058) | (0.155) | (0.086) | (0.068) | (0.094) | (0.143) |
| All recommended vaccinations | -0.085* | 0.034 | 0.058 | -0.050 | -0.179** | -0.257** | -0.088* | 0.277 | 0.025 | -0.151** | -0.208** | -0.317** |
|  | (0.044) | (0.094) | (0.069) | (0.061) | (0.075) | (0.119) | (0.048) | (0.171) | (0.071) | (0.072) | (0.092) | (0.135) |
| Dependency ratio | -0.053* | -0.077 | -0.064 | -0.045 | -0.021 | -0.074 | -0.054* | -0.203** | -0.073 | -0.040 | -0.023 | 0.088 |
|  | (0.027) | (0.059) | (0.049) | (0.037) | (0.037) | (0.057) | (0.031) | (0.080) | (0.052) | (0.039) | (0.064) | (0.088) |
| BMI | 0.027*** | 0.035** | 0.028** | 0.015 | 0.013 | 0.021 | 0.027*** | 0.021 | 0.026*** | 0.027* | 0.015 | 0.064** |
|  | (0.008) | (0.018) | (0.012) | (0.011) | (0.011) | (0.021) | (0.007) | (0.017) | (0.010) | (0.014) | (0.012) | (0.025) |
| Mother currently working | -0.065 | -0.012 | -0.068 | -0.025 | -0.082 | -0.230* | -0.116*** | -0.068 | -0.072 | -0.165** | -0.125 | -0.154 |
|  | (0.043) | (0.097) | (0.063) | (0.063) | (0.076) | (0.119) | (0.045) | (0.114) | (0.063) | (0.066) | (0.092) | (0.156) |
| Maternal education | 0.063 | 0.235* | 0.069 | 0.017 | -0.059 | -0.001 | 0.028 | 0.268 | 0.094 | 0.060 | -0.158* | -0.231 |
|  | (0.053) | (0.138) | (0.088) | (0.074) | (0.090) | (0.133) | (0.058) | (0.172) | (0.093) | (0.079) | (0.094) | (0.181) |
| Father’s education | 0.161** | 0.224 | 0.334*** | 0.115 | 0.059 | -0.091 | 0.100 | 0.151 | 0.124 | 0.043 | 0.067 | 0.119 |
|  | (0.063) | (0.190) | (0.120) | (0.111) | (0.095) | (0.168) | (0.069) | (0.161) | (0.102) | (0.088) | (0.109) | (0.189) |
| Wealth index | 0.210*** | 0.111 | 0.247*** | 0.208*** | 0.198*** | 0.319** | 0.189*** | 0.085 | 0.110*** | 0.150*** | 0.207*** | 0.238** |
|  | (0.036) | (0.071) | (0.049) | (0.049) | (0.064) | (0.127) | (0.031) | (0.058) | (0.037) | (0.043) | (0.063) | (0.094) |
| Improved water use | 0.028 | -0.012 | 0.042 | 0.046 | -0.002 | -0.000 | 0.074* | 0.092 | 0.045 | 0.055 | 0.018 | 0.174 |
|  | (0.044) | (0.100) | (0.069) | (0.067) | (0.079) | (0.137) | (0.044) | (0.108) | (0.080) | (0.066) | (0.077) | (0.106) |
| Improved sanitation | -0.064 | -0.057 | -0.099 | -0.046 | 0.100 | -0.130 | -0.052 | 0.137 | 0.012 | 0.051 | -0.124 | -0.190 |
|  | (0.081) | (0.189) | (0.121) | (0.138) | (0.146) | (0.260) | (0.061) | (0.124) | (0.094) | (0.083) | (0.085) | (0.201) |
| Community sanitation | 0.467*** | 0.562*** | 0.533*** | 0.514*** | 0.311 | 0.567 | 0.197** | 0.200 | 0.267* | 0.239* | 0.282* | 0.231 |
|  | (0.112) | (0.218) | (0.150) | (0.168) | (0.247) | (0.355) | (0.100) | (0.235) | (0.155) | (0.123) | (0.152) | (0.300) |
| North West region | -0.244*** | -0.159 | -0.229** | -0.290*** | -0.269*** | -0.182 | -0.032 | -0.140 | -0.094 | -0.074 | -0.050 | 0.097 |
|  | (0.049) | (0.116) | (0.113) | (0.083) | (0.070) | (0.119) | (0.050) | (0.110) | (0.070) | (0.065) | (0.082) | (0.112) |
| North East region | -0.269*** | -0.412*** | -0.121 | -0.285*** | -0.205** | -0.179 | -0.127 | -0.499** | -0.304*** | -0.149** | -0.059 | 0.210 |
|  | (0.093) | (0.138) | (0.127) | (0.078) | (0.089) | (0.142) | (0.090) | (0.197) | (0.075) | (0.061) | (0.081) | (0.161) |
| South West region | 0.107 | 0.122 | 0.107 | 0.012 | 0.199* | 0.022 | -0.024 | 0.253 | 0.112 | -0.040 | -0.087 | -0.149 |
|  | (0.081) | (0.117) | (0.128) | (0.122) | (0.116) | (0.170) | (0.090) | (0.157) | (0.115) | (0.118) | (0.113) | (0.197) |
| Constant | -1.807*** | -4.273*** | -3.103*** | -1.571*** | -0.344 | 0.781 | -1.823*** | -4.033*** | -3.135*** | -1.995*** | -0.335 | -0.289 |
|  | (0.179) | (0.451) | (0.328) | (0.270) | (0.278) | (0.493) | (0.180) | (0.441) | (0.244) | (0.285) | (0.305) | (0.546) |
| N | 3407 | 3407 | 3407 | 3407 | 3407 | 3407 | 3590 | 3590 | 3590 | 3590 | 3590 | 3590 |
| R-sq | 0.190 | 0.046 | 0.091 | 0.126 | 0.159 | 0.107 | 0.144 | 0.049 | 0.077 | 0.112 | 0.122 | 0.078 |
| adj. R-sq | 0.185 | 0.041 | 0.086 | 0.122 | 0.154 | 0.102 | 0.139 | 0.045 | 0.073 | 0.108 | 0.118 | 0.073 |
| Figures in brackets are standard errors; asterisks show level of significance ***= significant at 1% level, **=significant at 5% level and *=significant at 10% level. | | | | | | | | | | | | |

**Table 8: Unconditional RIF regression results for rural Cambodia 2005 - 2010**

|  | 2005 | | | | | | 2010 | | | | | |
| --- | --- | --- | --- | --- | --- | --- | --- | --- | --- | --- | --- | --- |
|  |  | Quantile regression - quantiles | | | | |  | Quantile regression - quantiles | | | | |
|  | OLS | 10^th^ | 25^th^ | 50^th^ | 75^th^ | 90^th^ | OLS | 10^th^ | 25^th^ | 50^th^ | 75^th^ | 90^th^ |
| Gender of child (0=male, 1=female) | 0.160*** | 0.259*** | 0.279*** | 0.136* | 0.075 | 0.109 | 0.025 | -0.044 | 0.054 | 0.050 | 0.035 | 0.028 |
|  | (0.046) | (0.095) | (0.088) | (0.073) | (0.061) | (0.109) | (0.048) | (0.116) | (0.066) | (0.062) | (0.064) | (0.104) |
| Age of child (months) | -0.025*** | -0.012*** | -0.018*** | -0.022*** | -0.032*** | -0.046*** | -0.021*** | -0.019*** | -0.012*** | -0.016*** | -0.028*** | -0.039*** |
|  | (0.001) | (0.003) | (0.002) | (0.002) | (0.004) | (0.004) | (0.002) | (0.004) | (0.002) | (0.003) | (0.003) | (0.005) |
| Birth in hospital | -0.094 | -0.155 | -0.287** | -0.046 | 0.031 | 0.004 | 0.105* | -0.031 | 0.114* | 0.160** | 0.179** | 0.116 |
|  | (0.067) | (0.139) | (0.131) | (0.098) | (0.114) | (0.200) | (0.055) | (0.148) | (0.068) | (0.067) | (0.085) | (0.145) |
| Breastfeeding within hour of birth | 0.029 | 0.032 | 0.070 | -0.031 | 0.056 | 0.077 | 0.146*** | 0.243** | 0.172*** | 0.087 | 0.059 | 0.180 |
|  | (0.048) | (0.081) | (0.085) | (0.067) | (0.071) | (0.123) | (0.052) | (0.123) | (0.062) | (0.064) | (0.077) | (0.116) |
| Prenatal visits | 0.193*** | 0.129 | 0.235*** | 0.142* | 0.201** | 0.364*** | 0.096 | 0.375** | 0.172** | 0.109 | 0.027 | 0.053 |
|  | (0.049) | (0.093) | (0.067) | (0.074) | (0.083) | (0.117) | (0.065) | (0.183) | (0.072) | (0.094) | (0.120) | (0.173) |
| All recommended vaccinations | -0.104** | 0.026 | 0.010 | -0.107 | -0.192** | -0.312** | -0.119** | 0.256* | 0.015 | -0.182*** | -0.259** | -0.390*** |
|  | (0.049) | (0.125) | (0.099) | (0.067) | (0.076) | (0.131) | (0.055) | (0.144) | (0.059) | (0.070) | (0.111) | (0.142) |
| Dependency ratio | -0.042 | -0.040 | -0.013 | -0.032 | -0.028 | -0.091 | -0.060 | -0.291*** | -0.102* | -0.069 | -0.031 | 0.127 |
|  | (0.030) | (0.074) | (0.047) | (0.038) | (0.044) | (0.062) | (0.038) | (0.101) | (0.053) | (0.048) | (0.069) | (0.102) |
| BMI | 0.032*** | 0.038** | 0.032** | 0.018* | 0.017 | 0.033 | 0.030*** | 0.021 | 0.024** | 0.023** | 0.024 | 0.065*** |
|  | (0.009) | (0.016) | (0.015) | (0.010) | (0.015) | (0.022) | (0.008) | (0.020) | (0.011) | (0.010) | (0.016) | (0.024) |
| Mother currently working | -0.078 | 0.025 | -0.059 | -0.017 | -0.111 | -0.255** | -0.159*** | -0.033 | -0.072 | -0.181*** | -0.272*** | -0.281** |
|  | (0.048) | (0.101) | (0.071) | (0.065) | (0.083) | (0.129) | (0.052) | (0.129) | (0.076) | (0.060) | (0.096) | (0.141) |
| Maternal education | 0.053 | 0.235** | 0.040 | -0.004 | -0.034 | 0.068 | 0.009 | 0.178 | 0.041 | 0.050 | -0.141 | -0.163 |
|  | (0.059) | (0.112) | (0.096) | (0.084) | (0.089) | (0.147) | (0.064) | (0.152) | (0.083) | (0.070) | (0.113) | (0.146) |
| Father’s education | 0.218*** | 0.200 | 0.423*** | 0.182* | 0.127 | -0.072 | 0.084 | 0.059 | 0.088 | 0.063 | 0.170 | -0.075 |
|  | (0.070) | (0.145) | (0.125) | (0.095) | (0.106) | (0.183) | (0.076) | (0.165) | (0.126) | (0.096) | (0.117) | (0.217) |
| Wealth index | 0.161*** | 0.125 | 0.248*** | 0.162*** | 0.119* | 0.200 | 0.151*** | 0.109 | 0.154*** | 0.127*** | 0.117* | 0.178* |
|  | (0.043) | (0.078) | (0.076) | (0.061) | (0.070) | (0.130) | (0.041) | (0.100) | (0.055) | (0.044) | (0.068) | (0.098) |
| Improved water use | 0.035 | 0.017 | 0.025 | 0.050 | 0.003 | 0.045 | 0.068 | 0.096 | 0.042 | 0.079 | 0.026 | 0.158 |
|  | (0.049) | (0.106) | (0.089) | (0.084) | (0.089) | (0.141) | (0.050) | (0.148) | (0.054) | (0.065) | (0.087) | (0.127) |
| Improved sanitation | -0.058 | -0.114 | -0.135 | -0.036 | 0.019 | -0.101 | -0.074 | 0.107 | -0.053 | 0.023 | -0.141 | -0.184 |
|  | (0.092) | (0.204) | (0.177) | (0.109) | (0.140) | (0.276) | (0.070) | (0.172) | (0.089) | (0.088) | (0.124) | (0.159) |
| Community sanitation | 0.450*** | 0.605*** | 0.541*** | 0.504*** | 0.363 | 0.389 | 0.300** | 0.519* | 0.484*** | 0.307* | 0.269 | 0.159 |
|  | (0.134) | (0.204) | (0.189) | (0.167) | (0.255) | (0.391) | (0.124) | (0.281) | (0.167) | (0.159) | (0.189) | (0.299) |
| North West region | -0.254*** | -0.165* | -0.193** | -0.293*** | -0.284*** | -0.261** | -0.076 | -0.293** | -0.117 | -0.090 | -0.063 | -0.000 |
|  | (0.054) | (0.100) | (0.093) | (0.069) | (0.069) | (0.117) | (0.057) | (0.137) | (0.079) | (0.083) | (0.097) | (0.143) |
| North East region | -0.341*** | -0.528*** | -0.160 | -0.320*** | -0.237** | -0.199 | -0.152 | -0.481** | -0.297*** | -0.156* | -0.061 | 0.143 |
|  | (0.112) | (0.155) | (0.137) | (0.103) | (0.109) | (0.145) | (0.102) | (0.208) | (0.096) | (0.080) | (0.105) | (0.219) |
| South West region | 0.168* | 0.213 | 0.184 | 0.054 | 0.276** | 0.050 | -0.036 | 0.175 | 0.136 | -0.003 | 0.027 | -0.184 |
|  | (0.095) | (0.162) | (0.131) | (0.105) | (0.134) | (0.199) | (0.105) | (0.157) | (0.115) | (0.114) | (0.170) | (0.188) |
| Constant | -1.934*** | -4.340*** | -3.289*** | -1.676*** | -0.456 | 0.680 | -1.825*** | -3.746*** | -2.991*** | -1.925*** | -0.461 | -0.099 |
|  | (0.207) | (0.312) | (0.359) | (0.262) | (0.359) | (0.554) | (0.212) | (0.570) | (0.228) | (0.257) | (0.400) | (0.542) |
| N | 2722 | 2722 | 2722 | 2722 | 2722 | 2722 | 2649 | 2649 | 2649 | 2649 | 2649 | 2649 |
| R-sq | 0.174 | 0.044 | 0.081 | 0.110 | 0.146 | 0.119 | 0.137 | 0.053 | 0.078 | 0.105 | 0.120 | 0.086 |
| adj. R-sq | 0.169 | 0.038 | 0.075 | 0.104 | 0.140 | 0.113 | 0.132 | 0.047 | 0.072 | 0.099 | 0.114 | 0.080 |
| Figures in brackets are standard errors; asterisks show level of significance ***= significant at 1% level, **=significant at 5% level and *=significant at 10% level. | | | | | | | | | | | | |

**Table 9: Unconditional RIF regression results for urban Cambodia 2005 - 2010**

|  | 2005 | | | | | | 2010 | | | | | |
| --- | --- | --- | --- | --- | --- | --- | --- | --- | --- | --- | --- | --- |
|  |  | Quantile regression - quantiles | | | | |  | Quantile regression - quantiles | | | | |
|  | OLS | 10^th^ | 25^th^ | 50^th^ | 75^th^ | 90^th^ | OLS | 10^th^ | 25^th^ | 50^th^ | 75^th^ | 90^th^ |
| Gender of child (0=male, 1=female) | 0.126 | -0.128 | 0.034 | 0.052 | 0.374* | 0.165 | -0.201** | 0.201 | 0.131 | -0.183 | -0.369*** | -0.557** |
|  | (0.093) | (0.200) | (0.152) | (0.147) | (0.203) | (0.262) | (0.085) | (0.210) | (0.140) | (0.131) | (0.125) | (0.255) |
| Age of child (months) | -0.022*** | -0.015* | -0.018*** | -0.017*** | -0.025*** | -0.022** | -0.017*** | -0.015** | -0.010*** | -0.015*** | -0.019*** | -0.033*** |
|  | (0.003) | (0.008) | (0.005) | (0.004) | (0.007) | (0.009) | (0.003) | (0.006) | (0.004) | (0.004) | (0.005) | (0.009) |
| Birth in hospital | -0.026 | -0.177 | -0.229 | -0.035 | 0.350* | 0.011 | 0.061 | 0.162 | -0.029 | 0.007 | 0.074 | 0.208 |
|  | (0.120) | (0.347) | (0.261) | (0.181) | (0.192) | (0.187) | (0.136) | (0.361) | (0.178) | (0.155) | (0.123) | (0.175) |
| Breastfeeding within hour of birth | 0.219** | 0.261 | 0.116 | 0.129 | 0.308 | -0.050 | 0.120 | 0.233 | 0.176 | 0.092 | 0.146 | 0.107 |
|  | (0.096) | (0.200) | (0.170) | (0.134) | (0.207) | (0.276) | (0.090) | (0.204) | (0.150) | (0.141) | (0.141) | (0.228) |
| Prenatal visits | 0.190* | 0.207 | 0.219 | 0.115 | 0.228 | 0.472* | 0.118 | -0.237 | 0.058 | 0.190 | 0.212 | 0.346 |
|  | (0.112) | (0.318) | (0.189) | (0.157) | (0.239) | (0.243) | (0.169) | (0.363) | (0.296) | (0.282) | (0.223) | (0.370) |
| All recommended vaccinations | 0.109 | 0.120 | 0.369* | 0.396*** | -0.067 | -0.272 | 0.097 | 0.532* | 0.214 | 0.149 | -0.015 | 0.023 |
|  | (0.100) | (0.258) | (0.190) | (0.123) | (0.182) | (0.294) | (0.105) | (0.312) | (0.165) | (0.132) | (0.164) | (0.268) |
| Dependency ratio | -0.074 | -0.345* | -0.190 | -0.048 | -0.081 | 0.093 | 0.009 | 0.220 | 0.018 | -0.013 | -0.068 | 0.026 |
|  | (0.057) | (0.198) | (0.133) | (0.078) | (0.087) | (0.128) | (0.055) | (0.144) | (0.103) | (0.092) | (0.067) | (0.132) |
| BMI | 0.017 | 0.031 | 0.030 | 0.008 | 0.023 | -0.010 | 0.020 | 0.004 | 0.065*** | 0.011 | 0.023 | 0.078** |
|  | (0.015) | (0.027) | (0.023) | (0.024) | (0.039) | (0.043) | (0.013) | (0.043) | (0.024) | (0.022) | (0.022) | (0.038) |
| Mother currently working | -0.038 | -0.177 | -0.113 | -0.234 | 0.022 | 0.444 | 0.094 | 0.051 | -0.030 | 0.177 | 0.275** | 0.350 |
|  | (0.096) | (0.214) | (0.141) | (0.152) | (0.188) | (0.287) | (0.090) | (0.223) | (0.135) | (0.140) | (0.124) | (0.251) |
| Maternal education | 0.042 | -0.278 | 0.188 | -0.032 | -0.316 | 0.205 | 0.274 | 1.035 | 0.583* | 0.169 | 0.007 | -0.083 |
|  | (0.126) | (0.333) | (0.217) | (0.167) | (0.233) | (0.276) | (0.182) | (0.752) | (0.328) | (0.252) | (0.252) | (0.457) |
| Father’s education | -0.339** | 0.311 | -0.204 | -0.376* | -0.511* | -0.853 | 0.337 | 0.848 | 0.238 | 0.077 | 0.012 | -0.054 |
|  | (0.159) | (0.343) | (0.250) | (0.195) | (0.290) | (0.613) | (0.217) | (0.618) | (0.441) | (0.217) | (0.263) | (0.369) |
| Wealth index | 0.345*** | 0.265** | 0.399*** | 0.379*** | 0.270* | 0.283 | 0.269*** | 0.203** | 0.233*** | 0.202*** | 0.286*** | 0.362* |
|  | (0.064) | (0.132) | (0.105) | (0.109) | (0.151) | (0.176) | (0.047) | (0.095) | (0.073) | (0.066) | (0.094) | (0.194) |
| Improved water use | -0.130 | -0.073 | 0.175 | -0.130 | -0.319 | -0.617* | 0.115 | -0.082 | 0.060 | 0.019 | 0.032 | 0.072 |
|  | (0.111) | (0.326) | (0.178) | (0.152) | (0.217) | (0.318) | (0.106) | (0.311) | (0.172) | (0.135) | (0.152) | (0.281) |
| Improved sanitation | 0.078 | 0.044 | -0.048 | -0.026 | 0.389 | 0.182 | 0.300* | 0.085 | 0.273 | 0.161 | 0.203 | 0.659* |
|  | (0.175) | (0.378) | (0.286) | (0.256) | (0.330) | (0.389) | (0.156) | (0.333) | (0.255) | (0.193) | (0.168) | (0.396) |
| Community sanitation | 0.384* | 0.382 | 0.384 | 0.407 | 0.288 | 0.514 | -0.333 | 0.098 | -0.085 | 0.355 | -0.142 | -1.451* |
|  | (0.221) | (0.478) | (0.364) | (0.376) | (0.440) | (0.467) | (0.265) | (0.607) | (0.453) | (0.407) | (0.335) | (0.761) |
| North West region | -0.235* | -0.013 | 0.037 | -0.341* | -0.443 | -0.454 | 0.147 | 0.192 | -0.064 | 0.004 | 0.178 | 0.169 |
|  | (0.124) | (0.276) | (0.262) | (0.194) | (0.327) | (0.482) | (0.107) | (0.302) | (0.142) | (0.155) | (0.155) | (0.307) |
| North East region | -0.028 | 0.131 | 0.410 | -0.129 | -0.198 | -0.343 | 0.004 | -0.182 | -0.175 | 0.077 | 0.082 | 0.190 |
|  | (0.175) | (0.364) | (0.312) | (0.172) | (0.294) | (0.408) | (0.222) | (0.410) | (0.196) | (0.157) | (0.168) | (0.388) |
| South West region | -0.003 | -0.107 | 0.328 | -0.034 | -0.328 | -0.199 | 0.029 | 0.444 | -0.165 | 0.031 | 0.174 | -0.239 |
|  | (0.155) | (0.380) | (0.259) | (0.216) | (0.315) | (0.426) | (0.175) | (0.348) | (0.191) | (0.173) | (0.156) | (0.412) |
| Constant | -1.440*** | -3.464*** | -3.118*** | -1.241** | -0.300 | 1.203 | -2.495*** | -5.510*** | -4.797*** | -2.415*** | -1.255** | -0.684 |
|  | (0.356) | (0.774) | (0.507) | (0.549) | (0.902) | (0.933) | (0.431) | (1.282) | (0.793) | (0.677) | (0.631) | (1.061) |
| N | 685 | 685 | 685 | 685 | 685 | 685 | 941 | 941 | 941 | 941 | 941 | 941 |
| R-sq | 0.287 | 0.096 | 0.187 | 0.206 | 0.197 | 0.099 | 0.135 | 0.066 | 0.096 | 0.095 | 0.131 | 0.091 |
| adj. R-sq | 0.268 | 0.072 | 0.165 | 0.185 | 0.175 | 0.075 | 0.118 | 0.048 | 0.078 | 0.077 | 0.114 | 0.073 |
| Figures in brackets are standard errors; asterisks show level of significance ***= significant at 1% level, **=significant at 5% level and *=significant at 10% level. | | | | | | | | | | | | |

**Table 10: Unconditional RIF regression results for Cambodia 2010 - 2014**

|  | 2010 | | | | | | 2014 | | | | | |
| --- | --- | --- | --- | --- | --- | --- | --- | --- | --- | --- | --- | --- |
|  |  | Quantile regression - quantiles | | | | |  | Quantile regression - quantiles | | | | |
|  | OLS | 10^th^ | 25^th^ | 50^th^ | 75^th^ | 90^th^ | OLS | 10^th^ | 25^th^ | 50^th^ | 75^th^ | 90^th^ |
| Gender of child (0=male, 1=female) | -0.013 | -0.018 | 0.045 | 0.034 | -0.032 | -0.085 | 0.011 | 0.060 | -0.002 | 0.005 | -0.100 | -0.022 |
|  | (0.042) | (0.089) | (0.059) | (0.062) | (0.070) | (0.100) | (0.038) | (0.074) | (0.060) | (0.041) | (0.082) | (0.104) |
| Age of child (months) | -0.021*** | -0.016*** | -0.011*** | -0.016*** | -0.027*** | -0.038*** | -0.019*** | -0.006** | -0.008*** | -0.014*** | -0.026*** | -0.036*** |
|  | (0.001) | (0.004) | (0.002) | (0.002) | (0.003) | (0.006) | (0.001) | (0.002) | (0.002) | (0.002) | (0.003) | (0.003) |
| Birth in hospital | 0.107** | -0.001 | 0.163*** | 0.136** | 0.203** | 0.114 | 0.133** | -0.013 | 0.127 | 0.062 | 0.133 | 0.120 |
|  | (0.049) | (0.125) | (0.061) | (0.066) | (0.081) | (0.130) | (0.060) | (0.129) | (0.104) | (0.076) | (0.101) | (0.125) |
| Breastfeeding within hour of birth | 0.129*** | 0.217** | 0.128** | 0.076 | 0.048 | 0.135 | -0.051 | 0.010 | -0.022 | -0.073 | -0.097 | -0.062 |
|  | (0.044) | (0.108) | (0.056) | (0.056) | (0.070) | (0.122) | (0.041) | (0.076) | (0.057) | (0.052) | (0.073) | (0.115) |
| Prenatal visits | 0.088 | 0.375*** | 0.145** | 0.103 | 0.035 | 0.048 | 0.029 | 0.341** | 0.129 | 0.103 | -0.107 | 0.040 |
|  | (0.058) | (0.140) | (0.073) | (0.067) | (0.099) | (0.151) | (0.070) | (0.144) | (0.105) | (0.105) | (0.128) | (0.188) |
| All recommended vaccinations | -0.088* | 0.277** | 0.025 | -0.151** | -0.208** | -0.317** | -0.114** | -0.063 | -0.123* | -0.157*** | -0.229*** | -0.065 |
|  | (0.048) | (0.130) | (0.079) | (0.059) | (0.092) | (0.142) | (0.045) | (0.082) | (0.066) | (0.059) | (0.081) | (0.132) |
| Dependency ratio | -0.054* | -0.203** | -0.073 | -0.040 | -0.023 | 0.088 | -0.045 | -0.083 | -0.145*** | -0.075* | -0.069 | 0.096 |
|  | (0.031) | (0.092) | (0.046) | (0.047) | (0.051) | (0.084) | (0.032) | (0.060) | (0.045) | (0.045) | (0.053) | (0.077) |
| BMI | 0.027*** | 0.021 | 0.026** | 0.027** | 0.015 | 0.064*** | 0.037*** | 0.031*** | 0.047*** | 0.036*** | 0.041*** | 0.024** |
|  | (0.007) | (0.016) | (0.010) | (0.011) | (0.012) | (0.016) | (0.006) | (0.012) | (0.007) | (0.008) | (0.014) | (0.012) |
| Mother currently working | -0.116*** | -0.068 | -0.072 | -0.165*** | -0.125* | -0.154 | -0.048 | -0.053 | -0.074 | -0.013 | -0.111 | 0.044 |
|  | (0.045) | (0.112) | (0.064) | (0.058) | (0.068) | (0.133) | (0.040) | (0.074) | (0.067) | (0.064) | (0.069) | (0.103) |
| Maternal education | 0.028 | 0.268 | 0.094 | 0.060 | -0.158 | -0.231 | -0.034 | 0.015 | -0.016 | -0.049 | -0.146 | -0.028 |
|  | (0.058) | (0.165) | (0.081) | (0.065) | (0.098) | (0.172) | (0.062) | (0.131) | (0.083) | (0.071) | (0.098) | (0.092) |
| Father’s education | 0.100 | 0.151 | 0.124 | 0.043 | 0.067 | 0.119 | 0.063 | 0.282** | 0.119 | 0.044 | 0.038 | 0.136 |
|  | (0.069) | (0.189) | (0.105) | (0.092) | (0.100) | (0.188) | (0.066) | (0.116) | (0.105) | (0.064) | (0.116) | (0.146) |
| Wealth index | 0.205*** | 0.092 | 0.120*** | 0.163*** | 0.225*** | 0.259*** | 0.183*** | 0.087** | 0.142*** | 0.177*** | 0.195*** | 0.355*** |
|  | (0.033) | (0.062) | (0.041) | (0.043) | (0.056) | (0.099) | (0.027) | (0.043) | (0.033) | (0.037) | (0.059) | (0.079) |
| Improved water use | 0.074* | 0.092 | 0.045 | 0.055 | 0.018 | 0.174 | 0.095** | 0.029 | 0.098 | 0.125** | 0.077 | 0.108 |
|  | (0.044) | (0.115) | (0.068) | (0.063) | (0.067) | (0.123) | (0.040) | (0.080) | (0.061) | (0.060) | (0.077) | (0.093) |
| Improved sanitation | -0.052 | 0.137 | 0.012 | 0.051 | -0.124 | -0.190 | 0.103* | 0.035 | 0.065 | 0.182** | 0.176** | 0.001 |
|  | (0.061) | (0.130) | (0.099) | (0.091) | (0.105) | (0.194) | (0.054) | (0.077) | (0.077) | (0.088) | (0.082) | (0.123) |
| Community sanitation | 0.197** | 0.200 | 0.267* | 0.239* | 0.282 | 0.231 | 0.161* | 0.217 | 0.198 | 0.095 | 0.316** | 0.064 |
|  | (0.100) | (0.214) | (0.154) | (0.132) | (0.194) | (0.289) | (0.084) | (0.150) | (0.131) | (0.130) | (0.148) | (0.241) |
| North West region | -0.032 | -0.140 | -0.094 | -0.074 | -0.050 | 0.097 | 0.041 | -0.117 | 0.008 | 0.053 | 0.031 | 0.239** |
|  | (0.050) | (0.117) | (0.071) | (0.077) | (0.074) | (0.110) | (0.045) | (0.071) | (0.068) | (0.068) | (0.078) | (0.117) |
| North East region | -0.127 | -0.499*** | -0.304*** | -0.149 | -0.059 | 0.210 | 0.087 | 0.018 | 0.117 | 0.033 | 0.109 | 0.246* |
|  | (0.090) | (0.188) | (0.099) | (0.092) | (0.090) | (0.151) | (0.078) | (0.123) | (0.094) | (0.082) | (0.083) | (0.145) |
| South West region | -0.024 | 0.253* | 0.112 | -0.040 | -0.087 | -0.149 | 0.095 | 0.032 | 0.140 | 0.161* | 0.005 | -0.098 |
|  | (0.090) | (0.146) | (0.075) | (0.116) | (0.158) | (0.187) | (0.082) | (0.122) | (0.099) | (0.091) | (0.117) | (0.117) |
| Constant | -1.745*** | -3.998*** | -3.089*** | -1.933*** | -0.250 | -0.190 | -1.890*** | -4.027*** | -3.265*** | -2.062*** | -0.675* | 0.315 |
|  | (0.183) | (0.361) | (0.239) | (0.220) | (0.302) | (0.396) | (0.169) | (0.390) | (0.264) | (0.215) | (0.348) | (0.378) |
| N | 3590 | 3590 | 3590 | 3590 | 3590 | 3590 | 4240 | 4240 | 4240 | 4240 | 4240 | 4240 |
| R-sq | 0.144 | 0.049 | 0.077 | 0.112 | 0.122 | 0.078 | 0.126 | 0.026 | 0.059 | 0.089 | 0.108 | 0.079 |
| adj. R-sq | 0.139 | 0.045 | 0.073 | 0.108 | 0.118 | 0.073 | 0.123 | 0.022 | 0.055 | 0.085 | 0.104 | 0.075 |
| Figures in brackets are standard errors; asterisks show level of significance ***= significant at 1% level, **=significant at 5% level and *=significant at 10% level. | | | | | | | | | | | | |

**Table 11: Unconditional RIF regression results for rural Cambodia 2010 - 2014**

|  | 2010 | | | | | | 2014 | | | | | |
| --- | --- | --- | --- | --- | --- | --- | --- | --- | --- | --- | --- | --- |
|  |  | Quantile regression - quantiles | | | | |  | Quantile regression - quantiles | | | | |
|  | OLS | 10^th^ | 25^th^ | 50^th^ | 75^th^ | 90^th^ | OLS | 10^th^ | 25^th^ | 50^th^ | 75^th^ | 90^th^ |
| Gender of child (0=male, 1=female) | 0.025 | -0.044 | 0.054 | 0.050 | 0.035 | 0.028 | 0.020 | 0.051 | -0.009 | -0.008 | -0.036 | 0.101 |
|  | (0.048) | (0.118) | (0.067) | (0.063) | (0.078) | (0.140) | (0.044) | (0.067) | (0.064) | (0.063) | (0.091) | (0.116) |
| Age of child (months) | -0.021*** | -0.019*** | -0.012*** | -0.016*** | -0.028*** | -0.039*** | -0.019*** | -0.006** | -0.008*** | -0.013*** | -0.025*** | -0.040*** |
|  | (0.002) | (0.004) | (0.002) | (0.002) | (0.003) | (0.006) | (0.001) | (0.003) | (0.002) | (0.002) | (0.003) | (0.005) |
| Birth in hospital | 0.105* | -0.031 | 0.114 | 0.160* | 0.179* | 0.116 | 0.143** | 0.018 | 0.163* | 0.066 | 0.146 | 0.187 |
|  | (0.055) | (0.107) | (0.080) | (0.082) | (0.099) | (0.112) | (0.066) | (0.129) | (0.093) | (0.093) | (0.102) | (0.139) |
| Breastfeeding within hour of birth | 0.146*** | 0.243** | 0.172** | 0.087 | 0.059 | 0.180 | -0.042 | 0.015 | -0.031 | -0.087 | -0.060 | -0.020 |
|  | (0.052) | (0.105) | (0.071) | (0.063) | (0.094) | (0.121) | (0.048) | (0.071) | (0.070) | (0.067) | (0.088) | (0.130) |
| Prenatal visits | 0.096 | 0.375** | 0.172* | 0.109 | 0.027 | 0.053 | 0.035 | 0.246 | 0.112 | 0.121 | -0.111 | 0.065 |
|  | (0.065) | (0.170) | (0.104) | (0.085) | (0.112) | (0.139) | (0.078) | (0.188) | (0.108) | (0.112) | (0.140) | (0.201) |
| All recommended vaccinations | -0.119** | 0.256** | 0.015 | -0.182** | -0.259** | -0.390*** | -0.123** | -0.066 | -0.145* | -0.172** | -0.265*** | -0.119 |
|  | (0.055) | (0.109) | (0.072) | (0.074) | (0.104) | (0.145) | (0.052) | (0.079) | (0.075) | (0.069) | (0.096) | (0.130) |
| Dependency ratio | -0.060 | -0.291*** | -0.102* | -0.069 | -0.031 | 0.127 | -0.031 | -0.053 | -0.155*** | -0.069 | -0.049 | 0.164 |
|  | (0.038) | (0.102) | (0.062) | (0.047) | (0.059) | (0.109) | (0.036) | (0.070) | (0.056) | (0.048) | (0.054) | (0.123) |
| BMI | 0.030*** | 0.021 | 0.024** | 0.023* | 0.024 | 0.065** | 0.043*** | 0.038*** | 0.055*** | 0.042*** | 0.045*** | 0.031* |
|  | (0.008) | (0.017) | (0.011) | (0.014) | (0.014) | (0.027) | (0.007) | (0.010) | (0.009) | (0.010) | (0.012) | (0.018) |
| Mother currently working | -0.159*** | -0.033 | -0.072 | -0.181** | -0.272*** | -0.281** | -0.069 | -0.064 | -0.075 | -0.043 | -0.159** | -0.019 |
|  | (0.052) | (0.116) | (0.067) | (0.072) | (0.078) | (0.132) | (0.047) | (0.070) | (0.064) | (0.060) | (0.081) | (0.129) |
| Maternal education | 0.009 | 0.178 | 0.041 | 0.050 | -0.141 | -0.163 | -0.029 | 0.076 | -0.033 | -0.010 | -0.075 | 0.101 |
|  | (0.064) | (0.138) | (0.075) | (0.070) | (0.110) | (0.168) | (0.069) | (0.117) | (0.081) | (0.090) | (0.088) | (0.152) |
| Father’s education | 0.084 | 0.059 | 0.088 | 0.063 | 0.170* | -0.075 | 0.068 | 0.257* | 0.129 | 0.084 | -0.001 | 0.066 |
|  | (0.076) | (0.158) | (0.112) | (0.109) | (0.102) | (0.215) | (0.074) | (0.146) | (0.092) | (0.098) | (0.129) | (0.171) |
| Wealth index | 0.164*** | 0.119 | 0.168*** | 0.138** | 0.127* | 0.194* | 0.154*** | 0.098 | 0.143*** | 0.118** | 0.139* | 0.273** |
|  | (0.044) | (0.097) | (0.063) | (0.056) | (0.077) | (0.109) | (0.036) | (0.068) | (0.041) | (0.051) | (0.072) | (0.114) |
| Improved water use | 0.068 | 0.096 | 0.042 | 0.079 | 0.026 | 0.158 | 0.114** | 0.056 | 0.098 | 0.159** | 0.044 | 0.135 |
|  | (0.050) | (0.092) | (0.065) | (0.077) | (0.079) | (0.149) | (0.046) | (0.067) | (0.069) | (0.062) | (0.081) | (0.120) |
| Improved sanitation | -0.074 | 0.107 | -0.053 | 0.023 | -0.141 | -0.184 | 0.088 | 0.007 | 0.016 | 0.212** | 0.219** | -0.016 |
|  | (0.070) | (0.158) | (0.101) | (0.086) | (0.110) | (0.220) | (0.060) | (0.113) | (0.092) | (0.085) | (0.090) | (0.147) |
| Community sanitation | 0.300** | 0.519** | 0.484*** | 0.307* | 0.269 | 0.159 | 0.214** | 0.228 | 0.294** | 0.069 | 0.283 | 0.117 |
|  | (0.124) | (0.236) | (0.183) | (0.167) | (0.191) | (0.313) | (0.098) | (0.173) | (0.132) | (0.145) | (0.172) | (0.231) |
| North West region | -0.076 | -0.293** | -0.117* | -0.090 | -0.063 | -0.000 | 0.015 | -0.092 | 0.016 | 0.033 | 0.007 | 0.140 |
|  | (0.057) | (0.136) | (0.061) | (0.068) | (0.089) | (0.117) | (0.052) | (0.085) | (0.069) | (0.067) | (0.078) | (0.142) |
| North East region | -0.152 | -0.481* | -0.297*** | -0.156** | -0.061 | 0.143 | 0.095 | 0.017 | 0.131 | 0.043 | 0.137 | 0.345** |
|  | (0.102) | (0.249) | (0.101) | (0.076) | (0.105) | (0.152) | (0.090) | (0.146) | (0.115) | (0.096) | (0.123) | (0.176) |
| South West region | -0.036 | 0.175 | 0.136 | -0.003 | 0.027 | -0.184 | 0.082 | 0.062 | 0.196 | 0.188* | 0.017 | -0.307** |
|  | (0.105) | (0.168) | (0.119) | (0.124) | (0.155) | (0.261) | (0.098) | (0.132) | (0.122) | (0.109) | (0.122) | (0.156) |
| Constant | -1.763*** | -3.701*** | -2.927*** | -1.872*** | -0.413 | -0.026 | -2.048*** | -4.140*** | -3.445*** | -2.282*** | -0.861** | 0.026 |
|  | (0.217) | (0.450) | (0.273) | (0.277) | (0.363) | (0.527) | (0.195) | (0.361) | (0.276) | (0.290) | (0.379) | (0.554) |
| N | 2649 | 2649 | 2649 | 2649 | 2649 | 2649 | 3080 | 3080 | 3080 | 3080 | 3080 | 3080 |
| R-sq | 0.137 | 0.053 | 0.078 | 0.105 | 0.120 | 0.086 | 0.121 | 0.025 | 0.059 | 0.078 | 0.102 | 0.080 |
| adj. R-sq | 0.132 | 0.047 | 0.072 | 0.099 | 0.114 | 0.080 | 0.116 | 0.019 | 0.053 | 0.072 | 0.097 | 0.074 |
| Figures in brackets are standard errors; asterisks show level of significance ***= significant at 1% level, **=significant at 5% level and *=significant at 10% level. | | | | | | | | | | | | |

**Table 12: Unconditional RIF regression results for urban Cambodia 2010 - 2014**

|  | 2010 | | | | | | 2014 | | | | | |
| --- | --- | --- | --- | --- | --- | --- | --- | --- | --- | --- | --- | --- |
|  |  | Quantile regression - quantiles | | | | |  | Quantile regression - quantiles | | | | |
|  | OLS | 10^th^ | 25^th^ | 50^th^ | 75^th^ | 90^th^ | OLS | 10^th^ | 25^th^ | 50^th^ | 75^th^ | 90^th^ |
| Gender of child (0=male, 1=female) | -0.201** | 0.201 | 0.131 | -0.183 | -0.369*** | -0.557** | -0.051 | 0.078 | 0.100 | -0.028 | -0.312** | -0.130 |
|  | (0.085) | (0.193) | (0.112) | (0.132) | (0.137) | (0.258) | (0.076) | (0.190) | (0.137) | (0.098) | (0.155) | (0.129) |
| Age of child (months) | -0.017*** | -0.015** | -0.010** | -0.015*** | -0.019*** | -0.033*** | -0.016*** | -0.001 | -0.008* | -0.015*** | -0.022*** | -0.017*** |
|  | (0.003) | (0.006) | (0.005) | (0.005) | (0.005) | (0.007) | (0.003) | (0.004) | (0.005) | (0.004) | (0.006) | (0.006) |
| Birth in hospital | 0.061 | 0.162 | -0.029 | 0.007 | 0.074 | 0.208 | -0.078 | -0.504 | 0.069 | 0.083 | 0.116 | -0.114 |
|  | (0.136) | (0.274) | (0.188) | (0.152) | (0.128) | (0.236) | (0.234) | (0.331) | (0.349) | (0.284) | (0.207) | (0.166) |
| Breastfeeding within hour of birth | 0.120 | 0.233 | 0.176 | 0.092 | 0.146 | 0.107 | -0.075 | -0.068 | 0.110 | -0.144 | -0.255 | -0.283* |
|  | (0.090) | (0.210) | (0.116) | (0.126) | (0.143) | (0.205) | (0.079) | (0.146) | (0.125) | (0.110) | (0.205) | (0.155) |
| Prenatal visits | 0.118 | -0.237 | 0.058 | 0.190 | 0.212 | 0.346 | 0.102 | 0.392 | 0.171 | 0.146 | -0.355 | 0.152 |
|  | (0.169) | (0.393) | (0.298) | (0.268) | (0.213) | (0.373) | (0.195) | (0.402) | (0.389) | (0.248) | (0.338) | (0.311) |
| All recommended vaccinations | 0.097 | 0.532** | 0.214 | 0.149 | -0.015 | 0.023 | -0.083 | -0.185 | 0.087 | -0.092 | -0.122 | -0.139 |
|  | (0.105) | (0.265) | (0.160) | (0.165) | (0.172) | (0.345) | (0.100) | (0.123) | (0.190) | (0.144) | (0.224) | (0.186) |
| Dependency ratio | 0.009 | 0.220 | 0.018 | -0.013 | -0.068 | 0.026 | -0.175** | -0.176 | -0.237* | -0.229** | -0.084 | -0.102 |
|  | (0.055) | (0.141) | (0.097) | (0.099) | (0.058) | (0.117) | (0.074) | (0.146) | (0.142) | (0.099) | (0.141) | (0.108) |
| BMI | 0.020 | 0.004 | 0.065*** | 0.011 | 0.023 | 0.078* | 0.006 | 0.007 | -0.004 | 0.002 | 0.008 | 0.000 |
|  | (0.013) | (0.040) | (0.021) | (0.023) | (0.022) | (0.040) | (0.011) | (0.024) | (0.020) | (0.016) | (0.025) | (0.019) |
| Mother currently working | 0.094 | 0.051 | -0.030 | 0.177 | 0.275** | 0.350* | 0.098 | 0.005 | 0.018 | 0.126 | 0.150 | 0.234* |
|  | (0.090) | (0.230) | (0.145) | (0.158) | (0.132) | (0.195) | (0.081) | (0.154) | (0.149) | (0.128) | (0.194) | (0.142) |
| Maternal education | 0.274 | 1.035* | 0.583** | 0.169 | 0.007 | -0.083 | 0.050 | -0.068 | 0.089 | 0.164 | -0.041 | 0.008 |
|  | (0.182) | (0.556) | (0.260) | (0.231) | (0.190) | (0.436) | (0.193) | (0.473) | (0.330) | (0.302) | (0.448) | (0.221) |
| Father’s education | 0.337 | 0.848 | 0.238 | 0.077 | 0.012 | -0.054 | -0.181 | -0.303 | -0.247 | -0.189 | -0.259 | 0.013 |
|  | (0.217) | (0.804) | (0.419) | (0.256) | (0.197) | (0.363) | (0.214) | (0.356) | (0.366) | (0.351) | (0.442) | (0.182) |
| Wealth index | 0.292*** | 0.220* | 0.253*** | 0.220** | 0.311*** | 0.393** | 0.269*** | 0.175** | 0.314*** | 0.281*** | 0.248** | 0.221** |
|  | (0.051) | (0.128) | (0.060) | (0.091) | (0.106) | (0.164) | (0.045) | (0.083) | (0.074) | (0.069) | (0.106) | (0.087) |
| Improved water use | 0.115 | -0.082 | 0.060 | 0.019 | 0.032 | 0.072 | -0.026 | 0.027 | 0.116 | 0.035 | 0.171 | -0.165 |
|  | (0.106) | (0.246) | (0.149) | (0.159) | (0.167) | (0.225) | (0.088) | (0.152) | (0.135) | (0.121) | (0.155) | (0.221) |
| Improved sanitation | 0.300* | 0.085 | 0.273 | 0.161 | 0.203 | 0.659* | 0.494*** | 0.928** | 0.719*** | 0.040 | 0.412* | 0.266 |
|  | (0.156) | (0.295) | (0.229) | (0.170) | (0.237) | (0.381) | (0.168) | (0.428) | (0.254) | (0.206) | (0.225) | (0.201) |
| Community sanitation | -0.333 | 0.098 | -0.085 | 0.355 | -0.142 | -1.451** | -0.134 | -0.029 | -0.613* | -0.121 | 0.171 | -0.133 |
|  | (0.265) | (0.636) | (0.397) | (0.400) | (0.372) | (0.704) | (0.261) | (0.458) | (0.341) | (0.292) | (0.404) | (0.447) |
| North West region | 0.147 | 0.192 | -0.064 | 0.004 | 0.178 | 0.169 | 0.173* | 0.009 | 0.090 | 0.170 | 0.159 | 0.101 |
|  | (0.107) | (0.268) | (0.152) | (0.111) | (0.146) | (0.254) | (0.103) | (0.177) | (0.147) | (0.147) | (0.220) | (0.208) |
| North East region | 0.004 | -0.182 | -0.175 | 0.077 | 0.082 | 0.190 | 0.089 | 0.284 | 0.236 | 0.124 | -0.076 | -0.088 |
|  | (0.222) | (0.350) | (0.234) | (0.196) | (0.135) | (0.269) | (0.177) | (0.189) | (0.188) | (0.167) | (0.166) | (0.163) |
| South West region | 0.029 | 0.444 | -0.165 | 0.031 | 0.174 | -0.239 | 0.167 | 0.044 | 0.092 | -0.090 | 0.215 | 0.313 |
|  | (0.175) | (0.299) | (0.222) | (0.167) | (0.196) | (0.259) | (0.153) | (0.174) | (0.211) | (0.188) | (0.260) | (0.243) |
| Constant | -2.384*** | -5.426*** | -4.701*** | -2.331*** | -1.137* | -0.535 | -1.142** | -3.068*** | -2.283*** | -1.063* | 0.080 | 0.835 |
|  | (0.433) | (1.310) | (0.727) | (0.679) | (0.607) | (0.882) | (0.464) | (0.726) | (0.661) | (0.641) | (0.826) | (0.677) |
| N | 941 | 941 | 941 | 941 | 941 | 941 | 1160 | 1160 | 1160 | 1160 | 1160 | 1160 |
| R-sq | 0.135 | 0.066 | 0.096 | 0.095 | 0.131 | 0.091 | 0.129 | 0.052 | 0.085 | 0.104 | 0.086 | 0.064 |
| adj. R-sq | 0.118 | 0.048 | 0.078 | 0.077 | 0.114 | 0.073 | 0.115 | 0.037 | 0.070 | 0.089 | 0.072 | 0.049 |
| Figures in brackets are standard errors; asterisks show level of significance ***= significant at 1% level, **=significant at 5% level and *=significant at 10% level. | | | | | | | | | | | | |
