# Supplementary material for: What Explains Cambodia’s Success in Reducing Child Stunting-2000-2014?
Source: PLoS One. 2016 Sep 20;11(9):e0162668. doi: 10.1371/journal.pone.0162668 (PMC5029902; doi:10.1371/journal.pone.0162668)
Supplement: S3 Appendix — (DOCX) [file pone.0162668.s003.docx]

**S3 Appendix: Demographic and Health Surveys for Cambodia – Observations with missing data**

The analysis in this paper uses data from four rounds of Demographic and Health Surveys for Cambodia – for the years 2000, 2005, 2010 and 2014. The dataset from each round has observations with missing data on child HAZ scores (the dependent variable used in our model) and certain covariates. Table-1 below shows the percentage of observations with missing data by variable and survey year.

**Table-1: Percentage of missing observations by variable and survey year in Cambodia DHS datasets**

|  | **2000** | **2005** | **2010** | **2014** |
| --- | --- | --- | --- | --- |
| Children HAZ score | 57.27% | 54.21% | 52.77% | 37.03% |
| Gender (% of female children) | ^-^ | ^-^ | ^-^ | ^-^ |
| Age in months | 10.95% | 7.18% | 5.00%^-^ | 2.71% |
| Hospital birth (%) | ^-^ | ^-^ | ^-^ | ^-^ |
| Children breastfed within 1 hour of birth (%) | ^-^ | ^-^ | ^-^ | ^-^ |
| Mothers receiving pre-natal visits (%) | ^-^ | ^-^ | ^-^ | ^-^ |
| Children receiving recommended vaccinations (WHO) (%) | ^-^ | ^-^ | ^-^ | ^-^ |
| Dependency ratio | ^-^ | ^-^ | ^-^ | ^-^ |
| Mother’s BMI | 50.32% | 50.04% | 49.68% | 34.42% |
| Mothers currently working (%) | ^-^ | ^-^ | ^-^ | ^-^ |
| Mothers attending any school (%) | ^-^ | ^-^ | ^-^ | ^-^ |
| Fathers attending any school (%) | ^-^ | ^-^ | ^-^ | ^-^ |
| Rural household (%) | ^-^ | ^-^ | ^-^ | ^-^ |
| Continuous wealth index | 0.82% | 1.10%^-^ | 0.67% | 0.46% |
|  |  |  |  |  |
| Observations with at least one missing variable | 57.64% | 54.38% | 52.98% | 37.32% |

Note: DHS Surveys of 2000, 2005, 2010, 2014.

The observations with missing data were deleted from the analysis. The deletion of observations with missing data could introduce bias in our parameter estimates if the characteristics of the households included in the analysis were significantly different from the characteristics of households excluded from the analysis. Table -2 below compares the characteristics the full sample with the characteristics of the sub-sample with complete information. We find no significant differences in the characteristics the full sample and the sub-sample with complete information. This supports our assumption that observations with missing data occur randomly.

**Table-2: DHS Cambodia datasets: Statistical differences between characteristics of all sample households and sub-sample of households with complete information**

| Survey year | **2000** | **2005** | **2010** | **2014** |
| --- | --- | --- | --- | --- |
| Gender (% of female children) | -0.01 | 0.02 | 0.00 | -0.01 |
| Hospital birth (%) | 0.00 | 0.00 | -0.01 | 0.03^†^ |
| Children breastfed within 1 hour of birth (%) | 0.00 | 0.01 | 0.02 | 0.02 |
| Mothers receiving pre-natal visits (%) | -0.04^‡^ | -0.01 | 0.00 | 0.00 |
| Children receiving recommended vaccinations (WHO) (%) | -0.13^‡^ | -0.04^‡^ | -0.01 | -0.02 |
| Dependency ratio | 0.08^‡^ | 0.10^‡^ | 0.04^†^ | -0.02 |
| Mothers currently working (%) | -0.02 | 0.05^‡^ | -0.01 | -0.03^†^ |
| Mothers attending any school (%) | 0.01 | 0.00 | 0.00 | 0.01 |
| Fathers attending any school (%) | 0.03^†^ | 0.01 | 0.02^†^ | -0.02 |
| Rural household (%) | -0.01 | 0.00 | -0.01 | -0.01 |

Note: The superscript symbols † and ‡ respective indicate a 0.05 and 0.01 significance difference based on unweighted samples. Source: Surveys of 2000, 2005, 2010, 2014.
